# Therapeutic Patient Education in Adults with Chronic Lower Limb Musculoskeletal Pain: A Scoping Review

**DOI:** 10.3390/healthcare14030290

**Published:** 2026-01-23

**Authors:** Carla Vanti, Michael Bianchini, Alessio Mantineo, Francesco Ballardin, Paolo Pillastrini

**Affiliations:** 1 Department of Biomedical and Neuromotor Sciences (DIBINEM), University of Bologna, via Massarenti 9, 40138 Bologna, Italy; francesco.ballardin@unibo.it (F.B.); paolo.pillastrini@unibo.it (P.P.); 2 Sol et Salus Hospital, 47922 Rimini, Italy; michaelbianchini95@gmail.com; 3 Villa Bellombra Hospital, 40132 Bologna, Italy; alessio20mantineo@gmail.com; 4 Unit of Occupational Medicine, IRCCS Azienda Ospedaliero-Universitaria di Bologna, via Pelagio Palagi 9, 40138 Bologna, Italy

**Keywords:** pain management, lower extremity, physical therapy modalities, rehabilitation, education

## Abstract

**Highlights:**

**What are the main findings?**

- Clinical research on Therapeutic Patient Education (TPE) in patients with chronic lower limb musculoskeletal pain has focused primarily on hip and knee osteoarthritis.
- TPE varies significantly across studies in content, delivery mode, providers, and duration.

**What are the implications of the main findings?**

- Clinical research lacks information on TPE in chronic ankle musculoskeletal conditions and in young adults.
- Future studies should report providers’ training and TPE amount.

**Abstract:**

**Background**: Conservative treatment of chronic musculoskeletal pain includes exercise, manual therapy, medications, physical agents/modalities, and Therapeutic Patient Education (TPE). Research on TPE has predominantly focused on spinal pain, so we do not know the extent and scope of clinical research in other areas, particularly lower extremities. This review aimed to map current research on this topic. **Methods**: We searched PubMed, PEDro, CINAHL, PsycINFO, and Cochrane Library up to 1 April 2024. We included RCTs on adults with chronic lower limb musculoskeletal pain, written in English, French, Spanish, or Italian. **Results**: Fifty-two records concerning knee osteoarthritis (n.33), hip and knee osteoarthritis (n.8), hip osteoarthritis (n.3), chronic knee pain (n.3), patellofemoral pain (n.3), and gluteal tendinopathy (n.2) were included. TPE was delivered through self-management, disease-specific information, pain education, and the management of physical activity, load, diet, stress, and sleep. Interventions were both individual- and group-based; delivery methods included in-person intervention, telephone/video calls, and web tools/apps. TPE combined with exercise seemed to be more effective than exercise alone, information/little education, or usual care. The effects of TPE as a stand-alone intervention appeared uncertain. **Conclusions**: There is considerable variability in TPE in terms of teaching topics, providers, administration methods, and dosage of interventions. Future studies should investigate the effects of TPE in young adult populations and in ankle conditions. They should also investigate the effects of TPE on pain intensity versus pain interference with activities, by deepening TPE effects on disability and quality of life.

## 1. Introduction

Chronic musculoskeletal pain is defined as pain in the musculoskeletal system that persists or recurs for longer than three months; according to the World Health Organization (WHO), 20–33% of the world’s population has some form of chronic musculoskeletal pain, translating to 1.75 billion people globally [1]. This condition causes great direct and indirect social costs [2].

Treating chronic pain involves multifactorial interventions targeting physical, psychological, emotional, lifestyle, and social aspects. Among interventions delivered to chronic pain patients, the physical approach still tends to prevail over the multidisciplinary approach, and little attention is paid to Therapeutic Patient Education (TPE) [3].

According to the WHO’s definition, TPE includes a multifaceted intervention tailored to address patients’ conditions and characteristics, with different delivery methods and educational goals [4]. Importantly, TPE differs from generic patient education or information provision, as it is grounded in a structured, patient-centered, and biopsychosocial framework aimed at empowering patients in self-management and decision-making. In fact, TPE can vary in its teaching strategy (e.g., lectures, discussions, information technology, written materials, audiotapes, videotapes, oral instruction, and demonstrations) and delivery method (individual or group instruction). It may also include teaching patients about their conditions, treatment options, medication management, diet and exercise recommendations, behavioral changes, and coping strategies for better health management [5].

With the paradigm change from a biomedical to a biopsychosocial approach in the last decades of the 1900s, the focus shifted from pathology to patients. In this context, TPE started to play a crucial role in the field of physiotherapy treatments, both for patients (to empower them) and for physiotherapists (to broaden their skills beyond providing passive or active procedures). Then, TPE began to be a recommended intervention for different chronic conditions, i.e., musculoskeletal chronic pain [6,7], chronic low back pain (LBP) [8,9], and tendinopathies [10].

In the last three decades, within the rehabilitative musculoskeletal field, two new TPE approaches emerged; these are Pain Neuroscience Education (PNE) and Psychologically Informed Physical Therapy (PIPT).

PNE is an approach to chronic pain management that aims to educate individuals about the complex nature of pain [11], as well as the ways in which the nervous system is involved in the experience of pain [12]. This educational strategy aims to help individuals understand the neurobiological and neurophysiological processes of pain [12,13], including emotions, beliefs, and past experiences. Patients can better understand pain, thereby changing the negative beliefs they have about it and their incorrect perceptions of it [13,14]. Current research suggests PNE in chronic musculoskeletal pain could increase patient knowledge about pain, produce cognitive changes, and have positive effects on pain, disability, kinesiophobia, catastrophizing, hypervigilance, anxiety, attitudes, and beliefs [6,15,16].

PIPT is a multimodal rehabilitation approach that integrates psychological principles and usual physiotherapy interventions, with the aim of addressing the psychological and cognitive aspects that can influence patient behavior and outcomes [17]. The main interventions include different strategies, such as graded exposure, graded activity, cognitive–behavioral therapy [17,18], acceptance and commitment-based physical therapy [19,20], mindfulness, and relaxation techniques [21]. PIPT interventions should be tailored to each patient and could be delivered, depending on the patients’ psychosocial factors and needs, by physical therapists and/or psychologists [22].

In the musculoskeletal field, PNE demonstrated a moderate effect on reducing pain (especially when combined with exercise or other interventions), disability, and kinesiophobia [6]. PIPT interventions when combined with physical therapy showed small effects on reducing pain, disability, kinesiophobia, and pain catastrophizing [23] and improved physical function [24].

A recent umbrella review on PNE in musculoskeletal chronic conditions [6] showed that LBP, and in general spinal pain, is the most studied. Indeed, of the sixteen reviews included, only three considered different conditions (e.g., fibromyalgia, osteoarthritis). Similar considerations could be made on PIPT [17,23,24].

Current research often discusses the effects of TPE on chronic musculoskeletal pain regardless of the specific condition, while most of the evidence comes from populations with spinal disorders. Furthermore, the young adult population is underrepresented in existing studies, potentially limiting the generalizability of the findings. While it is reasonable to consider the mechanisms underlying chronic spinal pain similar to those of lower limb pathologies, generalizing the effects of TPE to different clinical conditions is questionable.

Therefore, we decided to carry out a scoping review [25] to map the clinical research concerning TPE in chronic painful conditions of the lower extremities. Given the heterogeneity of interventions labeled as TPE [26], this review aims to describe and categorize educational components rather than evaluate a single, homogeneous TPE construct.

The objectives of this study do not address the clinical effectiveness of different TPE interventions, which should be the subject of a systematic review.

## 2. Materials and Methods

This review was drafted according to the Preferred Reporting Items for Systematic Review and Meta-Analysis—Scoping Review (PRISMA-ScR) [27]. The PRISMA-ScR checklist is reported in Table S1. The protocol of this scoping review is registered in the Open Science Framework website: https://osf.io/j97zy/overview (accessed on 28 October 2025).

### 2.1. Deviations from the Protocol

Unlike what has previously been reported in the protocol, no additional searches on Google Scholar or Web of Science were conducted because of limited resources.

### 2.2. Eligibility Criteria

The PCC [Population, Concept, and Context] of this review is reported in Table 1.

Clinical research studies were included in this review according to the following criteria:

- Randomized Controlled Trials (RCTs) written in English, Italian, French, or Spanish;
- Involving adult patients (age ≥ 18 years) with chronic musculoskeletal pain in lower extremities;
- Comparing TPE, alone or combined with other interventions, with other interventions or no treatment;
- Considering at least one of the main clinical outcomes, i.e., pain intensity, pain interference with activities, function, disability, and quality of life.
- Studies were excluded in cases of the following:
- Other study designs (e.g., non-controlled clinical trials, observational studies, etc.);
- Study population including patients with systemic disease or diffuse pain;
- Study population including patients with chronic spinal or upper limb musculoskeletal pain;
- Treatment including surgery interventions;
- Educational interventions limited to passive information provision (e.g., prerecorded audio/video material or pamphlet delivery) without any face-to-face or interactive educational component.

### 2.3. Information Sources

PubMed, PEDro, CINAHL, PsycINFO, and the Cochrane databases were searched up to 1 April 2024, without any time restrictions.

Additionally, reference lists of included studies and other relevant papers retrieved with the search strategy were hand-searched to identify further studies. Given the aim of this review, which was to map clinical research in the published literature, no search of gray or unpublished research was conducted.

### 2.4. Search Strategy

The detailed search strategy for each database is reported in Table S2.

### 2.5. Selection of Sources of Evidence

Records retrieved from the search were uploaded to Zotero 6.0.36 software for automatic deduplication. Eligibility of titles and abstracts was independently evaluated by two reviewers (MB and AM) using the selection form by Rayyan software. If at least one author considered an article eligible, full-text and additional materials were retrieved. Eligibility of full texts was independently evaluated by two reviewers (MB and AM), and disagreements were solved by the intervention of a third reviewer (FB).

### 2.6. Data Charting Process

Data were collected by a single reviewer using a specific data charting form. Then, a second reviewer checked the extracted data.

### 2.7. Data Items

The data extraction included details identifying each publication (author and year of publication), country, setting, sample size, participants’ characteristics (age, sex, and condition), interventions (according to the TIDieR checklist [28]), follow-up times, and outcome measures.

### 2.8. Data Management and Validation

Disagreements in charting data were solved by the intervention of a third reviewer (FB).

### 2.9. Outcomes and Prioritization

Given the aim of this review, we considered pain, function, disability, and quality of life as main outcomes.

### 2.10. Risk of Bias (ROB)

A ROB assessment was conducted to contextualize the methodological quality and internal validity of the mapped evidence, without using it to exclude studies or rank their results, nor to infer effectiveness. The ROB assessment was performed by the authors group using the revised Johanna Briggs Institute (JBI) checklist, which is an appraisal tool to evaluate methodological quality and risk of bias in a wide range of study types [29]. Scores (n. yes/n. total) ≤ 49% were considered to be a high risk of bias, scores between 50% and 69% were considered to be a medium risk of bias, and scores ≥ 70% were considered to be a low risk of bias [30]. Any disagreements between reviewers were resolved with a third reviewer (FB).

### 2.11. Data Synthesis

According to the wide range of populations and educational interventions searched, we hypothesized great heterogeneity among the studies. A narrative summary was provided with information presented in the text, tables, and graphs to summarize the characteristics of the included studies (authors, year of publication, country of origin, population and sample size, type of intervention and comparators, and outcomes) and key findings related to the scoping review questions.

### 2.12. Meta-Bias

Since no meta-analysis or quantitative synthesis was performed, no meta-bias assessments were planned.

### 2.13. Confidence in Cumulative Evidence

Evidence was not quantitatively synthesized.

No generative artificial intelligence (GenAI) was used in this paper.

## 3. Results

### 3.1. Literature Search and Selection

**Figure 1:**
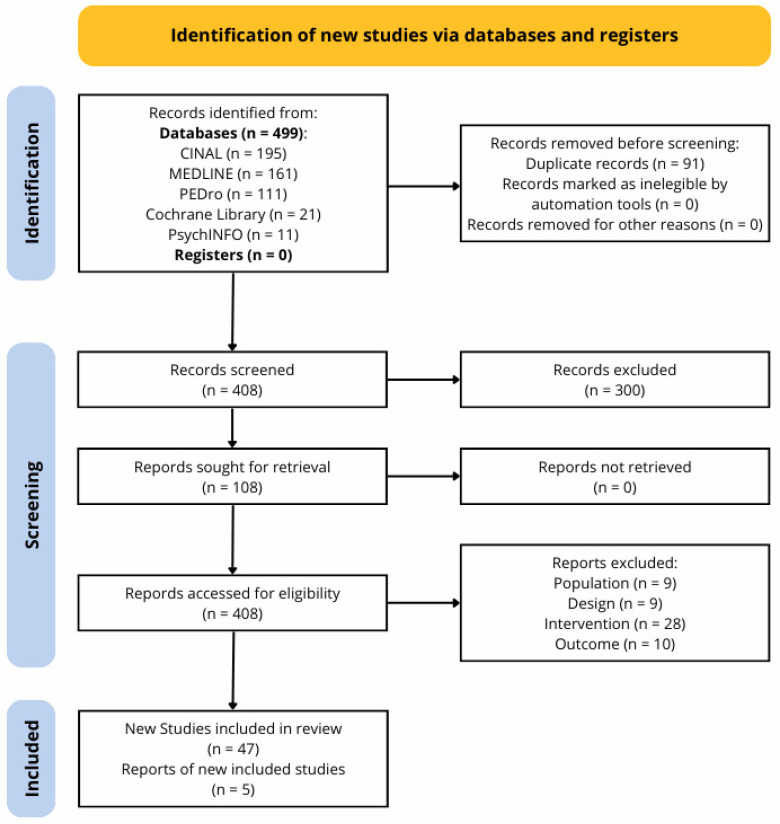
Flow chart of search and screening.

The flow chart (Figure 1) according to the PRISMA Flow Diagram tool [31] graphically represents the search and screening process.

The search retrieved 499 records. After automatic deduplication, 408 records remained, and titles and abstracts were screened for eligibility according to inclusion criteria. After the title and abstract screening process, 108 records were considered potentially eligible; then, full texts and Supplementary Materials were retrieved and screened for eligibility. After the screening process, 47 RCTs corresponding to 52 records were included [32,33,34,35,36,37,38,39,40,41,42,43,44,45,46,47,48,49,50,51,52,53,54,55,56,57,58,59,60,61,62,63,64,65,66,67,68,69,70,71,72,73,74,75,76,77,78,79,80,81,82,83]. No additional RCT was included from the reference lists. The excluded studies and the reasons for exclusion are reported in Table S3.

### 3.2. Characteristics of the Included Studies

General characteristics (first author, year of publication, country, and population) of the included studies are presented in Table 2.

**Figure 2:**
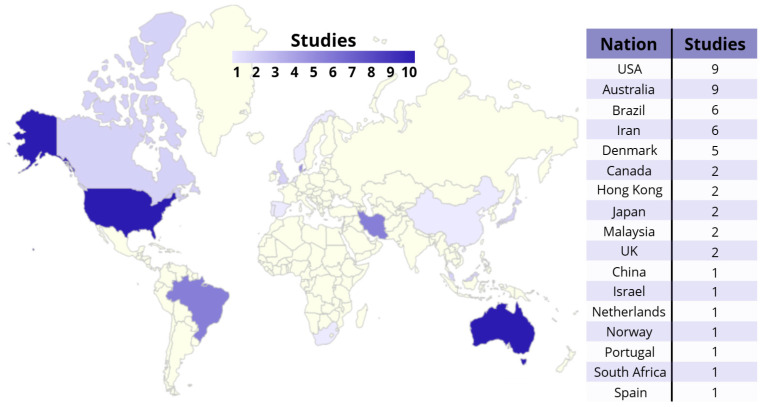
Geographic distribution of included studies.

The included studies were conducted in the USA (nine), Australia (nine, including one follow-up study), Brazil (six, including two follow-up studies), Iran (six), Denmark (five, including two follow-up studies), Canada (two), Hong Kong (two), Japan (two), Malaysia (two), UK (two), China (one), Israel (one), Netherlands (one), Norway (one), Portugal (one), South Africa (one), and Spain (one). They were published between 1997 and 2023 (median = 2017), with most published after 2010. A synthesis of the geographic distribution of the included studies is shown in Figure 2.

### 3.3. Brief Synthesis of Results

#### 3.3.1. Populations

Sample sizes ranged between 24 and 523 individuals (median = 113). Most studies calculated sample size based on pain or function outcomes, often resulting in small sample sizes.

The majority of the included studies involved older adult populations, with mean ages often exceeding 60 years, and reported a higher proportion of female participants. Only one study [51] investigated a young female population (mean age = 28 years) affected by patello-femoral pain.

**Figure 3:**
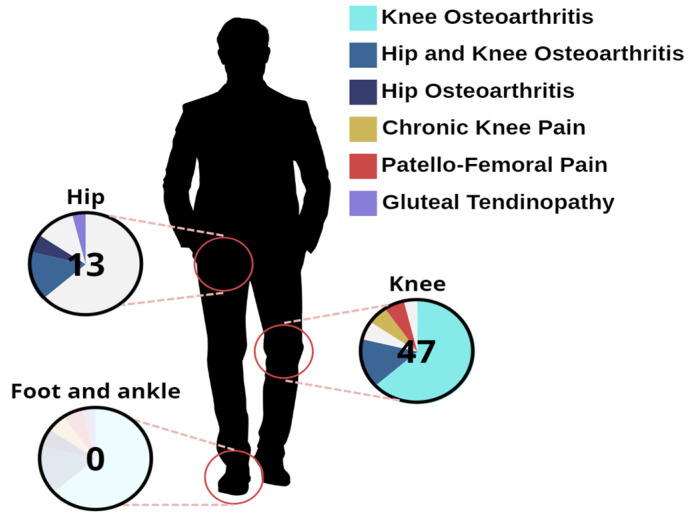
Number of studies on different chronic lower limb conditions.

The included studies investigated the effects of TPE interventions in different populations—hip and knee osteoarthritis (n.8), hip osteoarthritis alone (n.3), knee osteoarthritis alone (n.33, including 4 follow-up studies), chronic knee pain (n.3), patello-femoral pain (n.3), and gluteal tendinopathy (n.2, including 1 follow-up study). The pain conditions investigated in the included studies are graphically represented in Figure 3.

Only eight studies [41,46,51,53,56,62,63,77] focused on conditions other than osteoarthritis, and all but one [46] investigated TPE combined with exercise. Notably, no study addressed chronic foot or ankle pain, including conditions such as chronic ankle instability or Achilles tendinopathy.

#### 3.3.2. Interventions

In most studies, the educational intervention was delivered on the experimental group. Two of the included studies investigated the effect of TPE intervention both alone and in addition to the control intervention [32,33]; twenty studies investigated TPE intervention alone, and thirty investigated it in addition to other interventions (exercise, manual therapy, general physiotherapy, or medications). Educational interventions showed substantial heterogeneity in educational topics, providers, delivery modes, number of sessions, and intervention duration (Table S4).

- Topics—The results of this review show the great variability of education topics, e.g., specific pathology education, self-management education, physical activity education, load management, pain science education, diet education, stress management, and sleep education. Most interventions included multiple topics; however, stress management and sleep education are included in only two [51,58] and three [54,57,72] studies, respectively.
- Provider—Different providers delivered TPE intervention—in most cases, physiotherapists alone (19 studies) or with other professionals (10 studies), followed by multi-professional teams (7 studies) or other health professionals such as health educators (4 studies), clinical psychologists (2 studies), a Chinese medicine practitioner (1 study), and “researchers” not further specified (2 studies). In seven studies, the professional qualification of the providers was not specified. Fewer than half of the included studies reported that providers had specific training; others reported only the professional role of who provided the intervention and, in some cases, his/her years of expertise.
- Delivery mode—Delivery modes were diverse. Face-to-face intervention was the most used (44 studies), followed by phone/Internet-based calls (4 studies) and app- or web-based intervention (4 studies). They were provided on a group basis (26 studies), on an individual basis (20 studies), or on both group and individual bases (6 studies) sessions. One study [34] adopted multi-step intervention including three different steps: web education, coaching phone calls, and physiotherapy. Each participant proceeded to the next step every three months only if he/she had not yet reached the goal. This multi-step intervention tried to balance standardized web-delivered and individualized face-to-face interventions. None of the included studies used Artificial Intelligence (AI) to prepare and/or deliver educational programs.
- Sessions—The number of sessions delivered for TPE considerably varied, from one to 24; also, the time delivered for TPE was significantly different, from 30 min to 12 h. Some studies indicated the number of sessions without specifying their duration; others did not provide information on this topic.

#### 3.3.3. Outcome Measures

Pain was the most frequently reported outcome (45 studies, 83.69%), followed by function (26 studies, 50.00%), quality of life (13 studies, 25.00%), and disability (8 studies, 15.38%). A summary of outcome measures is presented in Table 3.

Only four RCTs [47,71,81,82] calculated sample size considering the quality of life as the main outcome.

#### 3.3.4. Risk of Bias

In line with the exploratory aim of this scoping review, the ROB findings are presented descriptively. Overall, the included RCTs showed moderate-to-high methodological quality. Specifically, only eight studies resulted in a high ROB, while the remaining studies were assessed as having a low (21 studies) or moderate (23 studies) ROB.

Blinding of participants and treatment providers was frequently absent or unclear; however, this should not be interpreted as a methodological shortcoming, as it is largely inherent to the nature of exercise- and education-based interventions, where blinding is often not feasible in practice. Outcome assessment was usually conducted in a standardized and reliable manner, although reliance on patient-reported outcome measures and limited reporting on assessor blinding introduced potential detection bias in several studies. Attrition was frequently reported, but inadequate analysis of dropouts and incomplete follow-up explanations remained common limitations. Statistical analyses were mostly appropriate for primary outcomes; however, pilot and feasibility studies often lacked sufficient power or comprehensive reporting for secondary outcomes.

A summary of ROB assessment is presented in Table 3, and a comprehensive ROB assessment for each study is reported in Table S5.

#### 3.3.5. Effectiveness of TPE Interventions

The results of the included studies for each outcome and each comparison are summarized in Table 4.

- Pain

Regarding pain reduction, when delivered as a stand-alone intervention, TPE generally demonstrated lower effectiveness compared to exercise [35,36,37]. When compared to information or minimal education, its effects ranged from similar [33,38,39,40,41,42,43] to superior [44,45,46]. TPE showed similar [42] to greater effects [44,47] compared to usual care and a similar effect compared to no intervention [48,49] or traditional Chinese medicine [50] at short-term follow-up.

When combined with exercise, TPE was associated with greater pain reduction compared to exercise alone [51,52], information/little education [53,54,55,56], usual care [57,58,59,60], heat therapy [61], and no intervention [62,63]. TPE combined with exercise also showed greater pain reduction compared to corticosteroid injection at short-term follow-up, whereas no differences were observed at long-term follow-up [62,63]. However, other studies did not find any statistically significant differences in pain reduction when comparing TPE plus exercise versus exercise alone [64], information/little education [41,65,66], usual care [67], inert injections [68,69], and no intervention [70].

Other studies reported greater pain reduction for TPE combined with different interventions versus medical care only [71,72] and delivery of information materials [33], whereas no differences were observed when comparing TPE combined with different interventions versus inert ultrasound [73] and physical therapy [74].

In summary, TPE appeared to be associated with favorable pain-related outcomes when combined with other interventions, particularly exercise. In contrast, findings for TPE delivered as a stand-alone intervention were inconsistent, and TPE alone appeared less effective when compared with exercise.

Detailed results for pain outcome are reported in Table S6.

- Function

Regarding function improvement, TPE as a stand-alone intervention mainly showed similar outcomes when compared to information/little education [38,39,40,41,43] and inferior outcomes compared to exercise [36]. However, two studies reported better outcomes for TPE alone compared to information/little education [46] and usual care [47].

TPE plus exercise mainly showed greater function improvement compared to exercise alone [51,52], information/little education [53,54,55,56], usual care [59,67], and heat therapy [61]. Nevertheless, other studies did not find statistically significant differences in function improvement when comparing TPE plus exercise versus exercise alone [64] and information/little education [41,66].

Two studies comparing TPE combined with different interventions versus medical care alone reported higher function improvement in the TPE group [71,72]. Other studies found no differences in function outcomes when comparing TPE plus different interventions versus inert ultrasound [73] and physical therapy [74].

Overall, TPE showed similar superior effects on function when combined with other interventions, particularly exercise; contradictory results emerged for TPE alone when compared to other interventions or usual care.

Detailed results for function outcome are reported in Table S7.

- Disability

Only eight studies reported disability outcomes [34,37,61,74,75,76,77,78,79]. TPE as a stand-alone intervention seemed to be less [37,76] to similarly [78] effective in reducing disability than exercise-based interventions, and when combined with exercise it showed greater effects than information/little education [34] and no additional effect when compared to exercise alone [77,79]. Education plus exercise appeared to be more effective than usual care [75] and heat therapy [61] in reducing disability.

Detailed results for disability outcome are reported in Table S8.

- Quality of life

As a stand-alone intervention, TPE seemed to have similar effects on improving quality of life compared to exercise [80], information/little education [39,40,46,81], and no intervention [49]. Furthermore, TPE alone appeared more effective than usual care in improving quality of life [47,82].

TPE combined with exercise showed similar effects on quality of life when compared to information/little education [66] and no intervention [70,83], while it showed little improvement in quality of life when compared to usual care [59], medical care [71], and corticosteroid injections [63].

Detailed results for the quality of life outcome are reported in Table S9.

## 4. Discussion

Clinical research on TPE in lower extremity conditions is mostly diffused in America and Australia, since fewer studies are conducted in Africa, Asia, and Europe.

Female and elderly patients mainly comprise the included populations. This could be explained by the prevalence of lower limb chronic musculoskeletal conditions that is higher in female gender, increases with age, and is frequently associated with hip and knee osteoarthritis [84,85,86]. However, ankle joints are also affected by chronic pain, even if this clinical condition seems underestimated [87].

This underestimation is confirmed by our review, in which most of the included studies were conducted on knee and hip osteoarthritis populations, and only eight of them focused on TPE effects in other lower limb conditions (chronic knee pain, patello-femoral pain, and gluteal tendinopathy). Notably, no RCT specifically investigated TPE interventions in chronic foot or ankle conditions, such as chronic ankle instability or Achilles tendinopathy, highlighting an important gap in the literature.

Overall, the methodological quality of the included studies was moderate to high. In accordance with the exploratory purpose of this review, the ROB assessment was performed to describe the quality of research in this area. Therefore, ROB results should be interpreted descriptively, not to draw conclusions about the reliability of the results.

A key finding of this review is the marked heterogeneity of TPE interventions. Even respecting the inclusion criteria adopted for this study, we notice that not all interventions labeled as TPE fully reflected the WHO’s conceptual framework. Given the limited reporting in some studies and the lack of objective criteria to determine full adherence, a conservative approach was adopted, and these interventions were retained. Educational content, delivery mode, providers, session number, and intervention duration varied widely, and TPE was delivered either alone or combined with other conservative treatments. This heterogeneity limits comparability across studies, precludes quantitative synthesis, and hinders the identification of consistent models or best practices.

### 4.1. Topic

Chronic pain is sustained by different factors including stress [88], poor sleep quality [89], poor diet [90], physical inactivity, and psychological and social factors. Given this complexity, effective TPE should begin with an individual assessment of factors contributing to each patient’s pain experience. Rather than prioritizing a single educational topic, tailored multimodal lifestyle interventions may better address individual needs [91]. These may include explanations of nociplastic [92] and phenotyping pain [93], clinical reasoning [94], integration of cognitive–behavioral strategies and pain education [95], the role of sleep [96], stress [97], physical activity and exercise [98], and nutrition [99]. Despite the multidimensional nature of chronic pain, stress management and sleep education were addressed in only a small minority of the included studies. Future research should therefore investigate the effectiveness of tailored, needs-based educational approaches and more systematically evaluate underrepresented domains such as stress and sleep, which may play a crucial role in patient outcomes.

### 4.2. Provider

According to the results of this review, different providers with different expertise were involved in TPE. The WHO’s 2023 TPE Guide [4] defines a “provider” as a healthcare professional (e.g., physiotherapist, occupational therapist, physician, nurse, psychologist, etc.) with skills to relate to each patient through patient-centered care and meaningful involvement of people with lived experience. Patient involvement in shared decision-making (clinical concept), self-assessment and self-efficacy, autonomy and empowerment (psychological concepts), and activation (behavioral concept) are the main goals of providers in the TPE field.

Providers should have specific knowledge and competencies in the fields of communication, clinical reasoning, assessing and treating biological, physical, psychological, and social factors, and the individualization and tailoring of intervention based on patient feedback [100]. However, fewer than half of the included studies reported whether providers had received specific training in therapeutic education. This lack of reporting limits the interpretability and reproducibility of TPE interventions.

As therapeutic education has evolved from a tissue- and disease-based approach toward a patient-centered approach, future studies should clearly describe not only the professional background of providers but also their specific training in TPE, as this may be a key determinant of intervention effectiveness.

### 4.3. Delivery Mode

Another important factor to take into consideration is the delivery mode of the TPE. As far as cost-effectiveness is concerned, one-to-one, face-to-face interventions provided by trained personnel can be more patient-specific but also more expensive than online group-based interventions [101]. Internet-based materials or apps are less expensive but may limit patients’ accessibility to these resources. Group sessions may foster peer support, whereas individual sessions allow for higher personalization at the cost of reduced social interaction. Some ongoing studies are investigating whether individual or group delivery methods influence clinical outcomes [102].

With the aim of individualizing TPE while saving clinicians’ time, recent approaches propose the use of Artificial Intelligence (AI). It has been highlighted that large language models, such as ChatGPT, Gemini, and Claude, may optimize the readability of patient information materials without compromising accuracy [103]. AI systems could potentially adapt educational content to individual literacy levels, answer patient-specific questions, and support the identification of relevant lifestyle factors [104]. The use of AI in TPE is in its infancy, and there are still some barriers and limitations in its use, such as the need to ensure the accuracy and reliability of the information provided and the need to guarantee empathetic communication [104].

### 4.4. Dosage

According to the current literature, it is not possible to identify a minimal session number, length, or frequency of TPE that could lead to an improvement for each outcome; therefore, it is very difficult to suggest a standardized dose [105]. For PNE interventions, a recent systematic review [106] reported the minimal dose for statistical effectiveness, in addition to exercise, as being between 150 and 200 total minutes of education, while for achieving a clinically important difference for pain, the minimal dose is higher (about 500 min). These preliminary results should be carefully considered, since that review included low to very low-quality studies. Similarly to education topics, the dose could be tailored to patients’ needs; more frequent sessions could be more effective (and more expensive) [107] but at the same time could reduce active patient engagement in the treatment. Future studies should clearly report the session number, duration, and total educational exposure to improve reproducibility and comparability.

### 4.5. Outcomes

Although pain is linked to various lifestyle factors, most studies focused primarily on pain and function, whereas quality of life and disability were less frequently assessed.

Overall, TPE appeared more effective when combined with exercise, particularly for pain and functional outcomes, compared to exercise alone, information-based interventions, or usual care. In contrast, results for TPE delivered as a stand-alone intervention were inconsistent and generally inferior to exercise-based approaches. These findings are consistent with previous reviews on LBP populations [7,8,108]. However, unlike some evidence suggesting benefits of education alone in LBP, the present review indicates that TPE may be insufficient as a single intervention for lower extremity conditions. This discrepancy may reflect differences in pain beliefs and psychosocial profiles across conditions; LBP is often associated with clinically relevant unhelpful beliefs [109], so education alone could have a higher effect in LBP than in other conditions.

The greater effects observed when TPE is combined with exercise may be related to improvements in pain interference rather than in pain intensity [58], suggesting that TPE may facilitate better coping, acceptance, and participation in daily activities. This is confirmed by other studies, which found a higher effect on disability than on pain reduction [7]. Further studies are needed to deeply understand if and how much TPE can influence quality of life and disability.

### 4.6. Limitations

This scoping review focused exclusively on RCTs, excluding observational and non-controlled studies. Additionally, no search of gray literature or protocol databases was conducted due to the mentioned deviations from the registered protocol. Therefore, results of this scoping review refer only to interventional trials and should not be generalized to the entire body of literature on this topic.

Moreover, the decision to exclude studies other than in English, Italian, French, or Spanish languages could have led to a literature bias, since the entire layer of works in other European or Asian languages was excluded from this review. However, only four studies were excluded due to language.

Although most studies showed moderate-to-high methodological quality, a substantial number were rated as having moderate or high ROB; therefore, their results should be interpreted with caution. The substantial heterogeneity of TPE interventions in terms of topics, dosage, and providers, as well as combined and comparator interventions prevented us from making definitive conclusions on best practices.

## 5. Conclusions

Although there is a wealth of literature on TPE in the treatment of chronic spinal pain, less is known about other chronic musculoskeletal conditions. This review highlights a lack of clinical studies regarding TPE on some frequent lower limb musculoskeletal conditions, such as chronic ankle instability or chronic tendinopathy (e.g., Achilles’ tendinopathy). Furthermore, TPE in younger adult populations is poorly investigated, and some contents of the TPE are underestimated, specifically stress management and sleep education.

TPE seems to be effective when combined with exercise, while its effects as a stand-alone intervention appear uncertain; more specifically, its effectiveness as a single treatment emerges as lower than that of therapeutic exercise. However, the available evidence suggests that TPE may exert more consistent effects on functional outcomes, disability, and patients’ ability to cope with pain, rather than on pain intensity alone, particularly when delivered alongside exercise.

Further high-quality research should investigate the effectiveness of TPE as a self-treatment method, also considering psychological and social factors. Future studies should also clarify not only the qualification but also the specific training of the providers, report the number of sessions and/or the total duration of the educational intervention, and better investigate quality of life and disability as outcome measures. Moreover, future studies should explicitly differentiate between pain intensity and pain interference with activities to better capture the potential clinical value of TPE beyond pain reduction alone. Finally, future research should investigate the integration of AI into interventions to understand whether AI helps patients improve their outcomes.

## Figures and Tables

**Table 1:** PCC of the review.

Population (P)	Adults with chronic musculoskeletal pain in lower extremities.
Concept (C)	Therapeutic Patient Education according to the WHO’s definition [4]. Interventions were included if they incorporated structured, interactive educational components.
Context (C)	Clinical, community, or mixed contexts.

**Table 2:** Characteristics of the studies.

FIRST AUTHOR	YEAR	NATION	POPULATION (OSTEOARTHRITIS)	SAMPLE SIZE	AGE	GENDER
Ackerman [81]	2012	Australia	Hip OA, Knee OA	126	Mean: 65.19 SD: NR	M: 38% F: 62%
Allen [44]	2010	USA	Hip OA, Knee OA	523	Mean: 60.1 SD: 10.4	M: 93% F: 7%
Hopman-Rock [70]	2000	Netherlands	Hip OA, Knee OA	120	Mean: 65.3 SD: 5.5	M: 0% F: 100%
Murphy [42]	2016	USA	Hip OA, Knee OA	193	Mean: 64.7 SD: 8.41	M: 38% F: 62%
Park [35]	2017	USA	Hip OA, Knee OA	112	Mean: 75.3 SD: 7.5	M: 24% F: 76%
Rini [48]	2021	USA	Hip OA, Knee OA	113	Mean: 67.62 SD: 9.45	M: 19% F: 81%
Saffari [82]	2018	Iran	Hip OA, Knee OA	120	Mean: NR SD: NR	M: 24% F: 76%
Saw [58]	2016	South Africa	Hip OA, Knee OA	74	Mean: 60.72 SD: 5.54	M: 19% F: 81%
Bennell [73]	2014	Australia	Hip OA	102	Mean: 63.56 SD: NR	M: 39% F: 61%
Olsen [43]	2022	Norway	Hip OA	101	Mean: 63.07 SD: NR	M: 79% F: 21%
Poulsen [33]	2013	Denmark	Hip OA	118	Mean: 64.63 SD: NR	M: 59% F: 41%
Ahmad [75]	2023	Malaysia	Knee OA	80	Mean: 65.43 SD: NR	NR
Allen [34]	2021	USA	Knee OA	345	Mean: 60 SD: 10.3	M: 85% F: 15%
Bandak [68]	2022	Denmark	Knee OA	206	Mean: 68.4 SD: NR	M: 54% F: 46%
Bennell [32]	2016	Australia	Knee OA	222	Mean: 63.42 SD: NR	M: 40% F: 60%
Bennell [74]	2017	Australia	Knee OA	168	Mean: 62.25 SD: NR	M: 37% F: 63%
Bennell [54]	2022	Australia	Knee OA	415	Mean: 64.74 SD: NR	M: 45% F: 55%
Bezalel [61]	2010	Israel	Knee OA	50	Mean: 75 SD: 5	M: 26% F: 74%
Brosseau [80]	2012	Canada	Knee OA	222	Mean: 63.4 SD: 8.6	M: 31% F: 69%
Chaharmahali [52]	2023	Iran	Knee OA	60	Mean: 54.13 SD: NR	M: 0% F: 100%
Cheung [50]	2019	Hong Kong	Knee OA	35	Mean: 62.14 SD: 5.93	M: 23% F: 79%
Cheung [65]	2020	Hong Kong	Knee OA	38	Mean: 74 SD: 7.03	M: 0% F: 100%
Coleman [47]	2012	Australia	Knee OA	147	Mean: 65 SD: 8	M: 25% F: 75%
Da Silva [55]	2015	Brazil	Knee OA	41	Mean: 58.5 SD: NR	M: 13% F: 87%
Ettinger [76]	1997	USA	Knee OA	439	Mean: 68.67 SD: NR	M: 30% F: 70%
Foo [57]	2020	Malaysia	Knee OA	300	Mean: 54.37 SD: NR	M: 17% F: 83%
Ganji [45]	2018	Iran	Knee OA	82	Mean: 64.96 SD: NR	NR
Henriksen [69]	2023	Denmark	Knee OA	206	Mean: 68.4 SD: NR	M: 54% F: 46%
Khachian [71]	2020	Iran	Knee OA	80	Mean: 58.5 SD: NR	M: 28% F: 72%
Marconcin [66]	2018	Portugal	Knee OA	80	Mean: 69.1 SD: 5.8	M: 0% F: 100%
Maurer [78]	1999	USA	Knee OA	113	Mean: 65.41 SD: NR	M: 58% F: 42%
Nagasawa [79]	2022	Japan	Knee OA	30	Mean: 74.2 SD: NR	M: 10% F: 90%
Nunez [83]	2006	Spain	Knee OA	100	Mean: 71.05 SD: NR	M: 29% F: 71%
Rabiei [64]	2023	Iran	Knee OA	54	Mean: 60.5 SD: 5.6	M: 59% F: 41%
Rezende [39]	2016	Brazil	Knee OA	228	Mean: NR SD: NR	M: 20% F: 80%
Rezende [38]	2017	Brazil	Knee OA	228	Mean: NR SD: NR	M: 20% F: 80%
Rezende [40]	2017	Brazil	Knee OA	228	Mean: NR SD: NR	M: 20% F: 80%
Rezende [72]	2021	Brazil	Knee OA	222	Mean: 63.5 SD: NR	M: 19% F: 81%
Skou [59]	2015	Denmark	Knee OA	100	Mean: 65.95 SD: NR	M: 49% F: 51%
Skou [60]	2016	Denmark	Knee OA	100	Mean: 65.95 SD: NR	M: 49% F: 51%
Song [36]	2022	China	Knee OA	40	Mean: 64.15 SD: 8.56	M: 0% F: 100%
Sullivan [67]	1998	USA	Knee OA	102	Mean: 72.96 SD: NR	M: 15% F: 85%
Taglietti [37]	2018	Brazil	Knee OA	60	Mean: 67.98 SD: NR	M: 32% F: 68%
Victor [49]	2005	UK	Knee OA	193	Mean: 63.13 SD: NR	M: 27% F: 73%
FIRST AUTHOR	YEAR	NATION	POPULATION (KNEE PAIN)	SAMPLE SIZE	AGE	GENDER
Bennell [53]	2017	Australia	Chronic Knee Pain	148	Mean: 61.15 SD: NR	M: 44% F: 56%
Jinnouchi [46]	2023	Japan	Chronic Knee Pain	46	Median: 69 IQR: 65–75	M: 11% F: 89%
Mecklenburg [56]	2018	USA	Chronic Knee Pain	162	Mean: 46 SD: 12	M: 60% F: 40%
Bagheri [51]	2021	Iran	Patello-femoral pain	30	Mean: 28.3 SD: 7.08	M: 0% F: 100%
Esculier [41]	2018	Canada	Patello-femoral pain	69	Mean: 30.8 SD: NR	M: 38% F: 62%
James [77]	2021	UK	Patello-femoral pain	24	Mean: 26.85 SD: NR	M: 25% F: 75%
FIRST AUTHOR	YEAR	NATION	POPULATION (TENDINOPATHY)	SAMPLE SIZE	AGE	GENDER
Mellor [62]	2018	Australia	Gluteal tendinopathy	204	Mean: 54.8 SD: 8.8	M: 19% F: 81%
Wilson [63]	2023	Australia	Gluteal tendinopathy	204	Mean: 54.8 SD: 8.8	M: 19% F: 81%

Legend of acronyms. F = females; M = males; NR = not reported; OA = osteoarthritis; SD = standard deviation; USA = United States of America; UK = United Kingdom.

**Table 3:** ROB assessment and outcome measures (pain, function, disability, and quality of life) for each study.

FIRST AUTHOR	YEAR	POPULATION (OSTEOARTHRITIS)	ROB: JBI SCORE (%)	OUTCOME MEASURES			
				PAIN	FUNCTION	DISABILITY	QUALITY OF LIFE
Ackerman [81]	2012	Hip OA, Knee OA	9/13 (69.23%)				HRQOL
Allen [44]	2010	Hip OA, Knee OA	7/13 (53.85%)	AIMS			
Hopman-Rock [70]	2000	Hip OA, Knee OA	4/13 (30.77%)	VAS			VAS-QOL
Murphy [42]	2016	Hip OA, Knee OA	9/13 (69.23%)	WOMAC-P			
Park [35]	2017	Hip OA, Knee OA	8/13 (61.54%)	WOMAC-P			
Rini [48]	2021	Hip OA, Knee OA	9/13 (69.23%)	AIMS			
Saffari [82]	2018	Hip OA, Knee OA	8/13 (61.54%)	EQ-VAS			SF-12, EQ-SD
Saw [58]	2016	Hip OA, Knee OA	5/13 (38.46%)	BPI			
Bennell [73]	2014	Hip OA	9/13 (69.23%)	VAS	WOMAC-F		
Olsen [43]	2022	Hip OA	9/13 (69.23%)	NRS	HOOS		
Poulsen [33]	2013	Hip OA	11/13 (84.62%)	NRS			
Ahmad [75]	2023	Knee OA	10/13 (76.92%)			KOOS	
Allen [34]	2021	Knee OA	10/13 (76.92%)	WOMAC-P	WOMAC-F	WOMAC-T	
Bandak [68]	2022	Knee OA	9/13 (69.23%)	KOOS-P			
Bennell [32]	2016	Knee OA	11/13 (84.62%)	NRS	WOMAC-F		
Bennell [74]	2017	Knee OA	10/13 (76.92%)	VAS	WOMAC-F		
Bennell [54]	2022	Knee OA	10/13 (76.92%)	NRS	WOMAC-F		
Bezalel [61]	2010	Knee OA	10/13 (76.92%)	WOMAC-P	WOMAC-F	WOMAC-T	
Brosseau [80]	2012	Knee OA	8/13 (61.54%)				SF-36
Chaharmahali [52]	2023	Knee OA	9/13 (69.23%)	VAS	WOMAC-F		
Cheung [50]	2019	Knee OA	9/13 (69.23%)	NRS			
Cheung [65]	2020	Knee OA	6/13 (46.15%)	NRS			
Coleman [47]	2012	Knee OA	11/13 (84.62%)	WOMAC-P	WOMAC-F		SF-36
Da Silva [55]	2015	Knee OA	10/13 (76.92%)	LEQUESNE INDEX	LEQUESNE INDEX		
Ettinger [76]	1997	Knee OA	9/13 (69.23%)	NRS	6MWT, LCT	SELFREPORT	
Foo [57]	2020	Knee OA	10/13 (76.92%)	KOOS-P			
Ganji [45]	2018	Knee OA	7/13 (53.85%)	VAS			
Henriksen [69]	2023	Knee OA	8/13 (61.54%)	KOOS-P			
Khachian [71]	2020	Knee OA	10/13 (76.92%)	KOOS-P	KOOS-ADL		KOOS-QOL
Marconcin [66]	2018	Knee OA	8/13 (61.54%)	KOOS-P	KOOS-ADL		KOOS-QOL
Maurer [78]	1999	Knee OA	6/13 (46.15%)			WOMAC	
Nagasawa [79]	2022	Knee OA	6/13 (46.15%)			JKOM	
Nunez [83]	2006	Knee OA	10/13 (76.92%)				SF-36
Rabiei [64]	2023	Knee OA	11/13 (84.62%)	WOMAC-P	WOMAC-F		
Rezende [39]	2016	Knee OA	7/13 (53.85%)	VAS	WOMAC		SF-36
Rezende [38]	2017	Knee OA	6/13 (46.15%)	VAS	WOMAC		SF-36
Rezende [40]	2017	Knee OA	7/13 (53.85%)		TUG, FTSTS		
Rezende [72]	2021	Knee OA	5/13 (38.46%)	WOMAC-P	WOMAC-F		
Skou [59]	2015	Knee OA	11/13 (84.62%)	KOOS-P	KOOS-ADL		KOOS-QOL
Skou [60]	2016	Knee OA	11/13 (84.62%)	VAS			
Song [36]	2022	Knee OA	10/13 (76.92%)	WOMAC-P	WOMAC-F		
Sullivan [67]	1998	Knee OA	3/13 (23.08%)	VAS	AIMS		
Taglietti [37]	2018	Knee OA	10/13 (76.92%)	VAS, WOMAC-P		WOMAC	
Victor [49]	2005	Knee OA	7/13 (53.85%)	WOMAC-P			
FIRST AUTHOR	YEAR	POPULATION (KNEE PAIN)	ROB: JBI SCORE (%)	OUTCOME MEASURES			
				PAIN	FUNCTION	DISABILITY	QUALITY OF LIFE
Bennell [53]	2017	Chronic Knee Pain	10/13 (76.92%)	NRS	WOMAC-F		
Jinnouchi [46]	2023	Chronic Knee Pain	9/13 (69.23%)	NRS	KOOS		EQ-5D
Mecklenburg [56]	2018	Chronic Knee Pain	7/13 (53.85%)	KOOS-P	KOOS-F		
Bagheri [51]	2021	Patello-femoral pain	10/13 (76.92%)	VAS	KOOS		
Esculier [41]	2018	Patello-femoral pain	9/13 (69.23%)	VAS	KOOS-ADL		
James [77]	2021	Patello-femoral pain	7/13 (53.85%)			KOOS-PF	
FIRST AUTHOR	YEAR	POPULATION (TENDINOPATHY)	ROB: JBI SCORE (%)	OUTCOME MEASURES			
				PAIN	FUNCTION	DISABILITY	QUALITY OF LIFE
Mellor [62]	2018	Gluteal tendinopathy	10/13 (76.92%)	NRS			
Wilson [63]	2023	Gluteal tendinopathy	10/13 (76.92%)				QALY

Legend of acronyms. AIMS (Arthritis Impact Measurement Scale); BPI (Brief Pain Inventory); EQ-5D (EuroQol Five-dimensional); EQ-VAS (EuroQol Visual Analogue Scale); F (Function); FTSTS (Five Times Sit To Stand Test); HOOS (Hip disability and Osteoarthritis Outcome Score); HRQOL (Health-Related Quality Of Life); JBI (Johanna Briggs Institute); JKOM (Japanese Knee Osteoarthritis Measure); KOOS (Knee Injury and Osteoarthritis Outcome Score); KOOS-PF (Knee Injury and Osteoarthritis Outcome Score for Patello-Femoral pain and osteoarthritis); LCT (Lifting and Carrying Test); NRS (Numeric Pain Rating Scale); OA (Osteoarthritis); P (Pain); QALY (Quality-Adjusted Life Year); QOL (Quality Of Life); ROB (Risk of Bias); SF-12 (Short Form 12 health survey questionnaire); SF-36 (Short Form 36 health survey questionnaire); 6MWT (6 Minutes Walking Test); T (Total); TUG (Time Up and Go); VAS (Visual Analogue Scale); WOMAC (Western Ontario and McMaster University Arthritis Index).

**Table 4:** Results for pain, function, disability and quality of life.

TPE INTERVENTION	VERSUS	CONTROL	PAIN	FUNCTION	DISABILITY	QUALITY OF LIFE
TPE alone	VS	Exercise	● [35,36,37]	● [36]	● [37,76]	
					● [78]	● [80]
TPE alone	VS	Information	● [33,38,39,40,41,42,43]	● [38,39,40,41,43]		● [39,40,46,81]
			● [44,45,46]	● [46]		
TPE alone	VS	Usual care	● [42]			
			● [44,47]	● [47]		● [47,82]
TPE alone	VS	No intervention	● [48,49]			● [49]
TPE alone	VS	Chinese medicine	● [50]			
TPE + Exercise	VS	Exercise	● [51,52]	● [51,52]	● [77,79]	● [66]
			● [64]	● [64]		
TPE + Exercise	VS	Information	● [53,54,55,56]	● [53,54,55,56]	● [34]	
			● [41,65,66]	● [41,66]		
TPE + Exercise	VS	Usual care	● [57,58,59,60]	● [59,67]	● [75]	● [59]
			● [67]			
TPE + Exercise	VS	Heat therapy	● [61]	● [61]	● [61]	
TPE + Exercise	VS	No intervention	● [62,63]			● [70,83]
			● [70]			
TPE + Exercise	VS	Sham intervention	● [68,69]			
TPE + Exercise	VS	Corticosteroid	● [62,63]			● [63]
TPE + Manual therapy	VS	Information	● [33]			
TPE + Physiotherapy	VS	Sham ultrasound	● [73]	● [73]		
TPE + Physiotherapy	VS	Physiotherapy	● [74]	● [74]		
TPE + Exercise + Medical care	VS	Medical care	● [71]	● [71]		● [71]
TPE + Medication	VS	Medication	● [72]	● [72]		

Legend of colors. ● = better results for TPE group; ● = similar results between TPE group and control group; ● = better results for control group. The references are reported in square brackets.

## Data Availability

The datasets generated and/or analyzed during the current study are available from the corresponding author upon reasonable request. The results presented in this study summarize all the data that were collected.

## Supplementary Materials

The following supporting information can be downloaded at https://www.mdpi.com/article/10.3390/healthcare14030290/s1, Table S1: PRISMA-ScR checklist; Table S2: Search strings; Table S3: Excluded records; Table S4: Synthesis of TPE interventions; Table S5: Risk of Bias assessment according to the revised JBI critical appraisal tool for RCTs; Table S6; Results on included studies on pain; Table S7: Results of included studies on function; Table S8: Results of included studies on disability; Table S9: Results of included studies on quality of life.

## Abbreviations

The following abbreviations are used in this manuscript:

AI	Artificial Intelligence
GenAI	Generative Artificial Intelligence
JBI	Johanna Briggs Institute
LBP	Low Back Pain
PCC	Population, Concept, Context
PNE	Pain Neuroscience Education
PIPT	Psychologically Informed Physical Therapy
PRISMA	Preferred Reporting Items for Systematic Review and Meta-Analysis
PRISMA-ScR	Preferred Reporting Items for Systematic Review and Meta-Analysis—Scoping Review
RCTs	Randomized Controlled Trials
ROB	Risk Of Bias
TIDieR	Template for Intervention Description and Replication
TPE	Therapeutic Patient Education
UK	United Kingdom
USA	United States of America
WHO	World Health Organization
