# Supplementary material for: Therapeutic Patient Education in Adults with Chronic Lower Limb Musculoskeletal Pain: A Scoping Review
Source: Healthcare (Basel). 2026 Jan 23;14(3):290. doi: 10.3390/healthcare14030290 (PMC12897465; doi:10.3390/healthcare14030290)
Supplement: Supplementary file 1 [file healthcare-14-00290-s001.zip › healthcare-4085082-supplementary.pdf]

Table S1. Preferred Reporting Items for Systematic reviews and Meta-Analyses extension for Scoping Reviews (PRISMA-ScR) Checklist.

SECTION	ITEM	PRISMA-ScR CHECKLIST ITEM	REPORTED ON PAGE
TITLE			
Title	1	Identify the report as a scoping review.	1
ABSTRACT			
Structured summary	2	Provide a structured summary that includes (as applicable): background, objectives, eligibility criteria, sources of evidence, charting methods, results, and conclusions that relate to the review questions and objectives.	1
INTRODUCTION			
Rationale	3	Describe the rationale for the review in the context of what is already known. Explain why the review questions/objectives lend themselves to a scoping review approach.	2,3
Objectives	4	Provide an explicit statement of the questions and objectives being addressed with reference to their key elements (e.g., population or participants, concepts, and context) or other relevant key elements used to conceptualize the review questions and/or objectives.	3
METHODS			
Protocol and registration	5	Indicate whether a review protocol exists; state if and where it can be accessed (e.g., a Web address); and if available, provide registration information, including the registration number.	3
Eligibility criteria	6	Specify characteristics of the sources of evidence used as eligibility criteria (e.g., years considered, language, and publication status), and provide a rationale.	3,4
Information sources	7	Describe all information sources in the search (e.g., databases with dates of coverage and contact with authors to identify additional sources), as well as the date the most recent search was executed.	4
Search	8	Present the full electronic search strategy for at least 1 database, including any limits used, such that it could be repeated.	Table S2
Selection of sources of evidence	9	State the process for selecting sources of evidence (i.e., screening and eligibility) included in the scoping review.	4

Data charting process	10	Describe the methods of charting data from the included sources of evidence (e.g., calibrated forms or forms that have been tested by the team before their use, and whether data charting was done independently or in duplicate) and any processes for obtaining and confirming data from investigators.	4
Data items	11	List and define all variables for which data were sought and any assumptions and simplifications made.	4
Critical appraisal of individual sources of evidence	12	If done, provide a rationale for conducting a critical appraisal of included sources of evidence; describe the methods used and how this information was used in any data synthesis (if appropriate).	4,5
Synthesis of results	13	Describe the methods of handling and summarizing the data that were charted.	5

SECTION	ITEM	PRISMA-ScR CHECKLIST ITEM	REPORTED ON PAGE
RESULTS			
Selection of sources of evidence	14	Give numbers of sources of evidence screened, assessed for eligibility, and included in the review, with reasons for exclusions at each stage, ideally using a flow diagram.	5,6
Characteristics of sources of evidence	15	For each source of evidence, present characteristics for which data were charted and provide the citations.	6-10
Critical appraisal within sources of evidence	16	If done, present data on critical appraisal of included sources of evidence (see item 12).	10-12
Results of individual sources of evidence	17	For each included source of evidence, present the relevant data that were charted that relate to the review questions and objectives.	12-14
Synthesis of results	18	Summarize and/or present the charting results as they relate to the review questions and objectives.	13,14
DISCUSSION			
Summary of evidence	19	Summarize the main results (including an overview of concepts, themes, and types of evidence available), link to the review questions and objectives, and consider the relevance to key groups.	15-17
Limitations	20	Discuss the limitations of the scoping review process.	17
Conclusions	21	Provide a general interpretation of the results with respect to the review questions and objectives, as well as potential implications and/or next steps.	17,18
FUNDING			
Funding	22	Describe sources of funding for the included sources of evidence, as well as sources of funding for the scoping review. Describe the role of the funders of the scoping review.	At the end of the text

From: Tricco AC, Lillie E, Zarin W, O'Brien KK, Colquhoun H, Levac D, et al. PRISMA Extension for Scoping Reviews (PRISMA-ScR): Checklist and Explanation. Ann Intern Med. ;169:467–473. doi: 10.7326/M18-0850

Table S2. Search strings.

Database	Search Strategy
PubMed	((((((((lower extremity[MeSH Terms]) OR (hip[Title/Abstract])) OR (knee[Title/Abstract])) OR (ankle[Title/Abstract])) OR (foot[Title/Abstract])) OR (thigh[Title/Abstract])) OR (leg[Title/Abstract])) AND ((((((((((((((((musculoskeletal pain[MeSH Terms]) OR (Osteoarthritis, Knee[MeSH Terms])) OR (Osteoarthritis, Hip[MeSH Terms])) OR (Piriformis Muscle Syndrome[MeSH Terms])) OR (Arthritis[MeSH Terms])) OR (Tendinopathy[MeSH Terms])) OR (Posterior Tibial Tendon Dysfunction[MeSH Terms])) OR (Fasciitis, Plantar[MeSH Terms])) OR (Achilles tendin*[Title/Abstract])) OR (patellar tendin*[Title/Abstract])) OR (osteoarthr*[Title/Abstract])) OR (tendin*[Title/Abstract])) OR (hallux valgus[Title/Abstract])) OR (hallux rigid*[Title/Abstract])) OR (tarsal tunnel syndrome[Title/Abstract])) OR (grea* trochanteric pain syndrome[Title/Abstract])) OR (GTPS[Title/Abstract])) OR (deep gluteal syndrome[Title/Abstract])) OR (piriformis syndrome[Title/Abstract]))) AND ((((((((((((patient education as topic[MeSH Terms]) OR (therapeutic patient Education[Title/Abstract]) OR (patient Education[Title/Abstract]) OR (therapeutic Education[Title/Abstract]) OR (Pain Neuroscience Education[Title/Abstract])) OR (PNE[Title/Abstract])) OR (Pain science Education[Title/Abstract])) OR (neuroscience education[Title/Abstract])) OR (psychological education[Title/Abstract])) OR (pain neurophysiological education[Title/Abstract])) OR (pain physiological education[Title/Abstract])) OR (neurobiology education[Title/Abstract])) OR (biology education[Title/Abstract])) OR (therapeutic neuroscience education[Title/Abstract])) OR ((((((((((((Psychotherapy[MeSH Terms]) OR (Psychotherapy[Title/Abstract])) OR (psychologically informed[Title/Abstract])) OR (psychologically-informed[Title/Abstract])) OR (cognitive behavioral[Title/Abstract])) OR (cognitive-behavioral[Title/Abstract])) OR (acceptance[Title/Abstract] AND commitment[Title/Abstract])) OR (mindfulness[Title/Abstract])) OR (Psycological based[Title/Abstract])) OR (psychological-based[Title/Abstract])) OR (psychological strategies[Title/Abstract])) AND (((((physical therapy specialty[MeSH Terms]) OR (rehabilitation[MeSH Terms])) OR (rehabilitation[Title/Abstract])) OR (Physiotherapy[Title/Abstract])) OR (Physical therapy[Title/Abstract]))))) AND (((((recovery of function[MeSH Terms]) OR (Pain[MeSH Terms])) OR (pain[Title/Abstract])) OR (disability[Title/Abstract])) OR (quality of life[Title/Abstrac]))t
Cochrane Library	(("lower extremity") OR ("hip") OR ("knee") OR ("ankle") OR ("foot")):ti,ab,kw AND (("musculoskeletal disorder") OR ("musculoskeletal pain")):ti,ab,kw AND (("patient education as topic") OR ("cognitive behavio* therapy") OR ("cognitive functional therapy") OR ("pain education") OR ("pain neuroscience education") OR ("Psychologically informed physical therapy")):ti,ab,kw AND (("Pain") OR ("disability") OR ("quality of life")):ti,ab,kw
PEDro	<u>BODY PART</u>: Thigh or hip / Lower leg or knee / Ankle and foot; <u>SUBDISCIPLINE</u>: Muscoloskeletal; <u>TOPIC</u>: Cronic pain; <u>METHOD</u>: RCT;<br><u>TITLE/ABSTRACT</u> : Multiple searches for the following terms: Patient education; Pain education; Psychologically informed; acceptance and commitment; Cognitive behavio* therapy; Cognitive functional therapy; Mindfulness

Database	Search Strategy
CINHAL and PsycINFO	(((MM "Lower Extremity+") OR (AB hip OR AB knee OR AB ankle OR AB foot OR AB thigh OR AB leg)) AND (((MM "Musculoskeletal Diseases+") OR (MM "Osteoarthritis, Knee") OR (MM "Osteoarthritis, Hip") OR (MM "Piriformis Syndrome") OR (MM "Patellofemoral Pain Syndrome") OR (MM "Arthritis+") OR (MM "Tendinopathy+") OR (MM "Patellar Tendinopathy") OR (MM "Posterior Tibial Tendon Dysfunction") OR (MM "Achilles Tendinopathy") OR (MM "Plantar Fasciitis") OR (MM "Tarsal Tunnel Syndrome")) OR AB tendin* OR AB osteoarthr* OR AB hallux valgus OR AB hallux rigid OR AB tarsal tunnel syndrome OR AB grea* trochanteric pain syndrome OR AB deep gluteal syndrome OR AB piriformis syndrome)) AND ((MM "Patient Education+") OR (AB Pain Neuroscience Education OR AB PNE OR AB Pain science Education OR AB neuroscience education OR AB psychological education OR AB pain neurophysiological education OR AB pain physiological education OR AB neurobiology education OR AB biology education OR AB therapeutic neuroscience education) OR ((AB Psychotherapy OR AB psychologically informed OR AB psychologically-informed OR AB cognitive behavioral OR AB cognitive-behavioral OR AB behavior OR AB acceptance commitment OR AB mindfulness OR AB Psycological based OR AB psychological-based OR AB psychological strategies) AND (AB rehabilitation OR AB Physiotherapy OR AB Physical therapy))) AND ((MM "Pain+") OR AB pain OR AB disability OR AB quality of life)

Table S3. Excluded records.

First Author	Year	Title	Exclusion reasons
Allen	2016	A combined patient and provider intervention for managing osteoarthritis in veterans: randomized clinical trial	Intervention
Allen	2017	Patient, provider, and combined interventions for managing osteoarthritis in primary care: a cluster randomized trial	Intervention
An	2021	Effects of preoperative telerehabilitation on muscle strength, range of motion, and functional outcomes in candidates for total knee arthroplasty: a single-blind randomized controlled trial	Intervention
Bennell	2018	Effects of internet-based pain coping skills training before home exercise for individuals with hip osteoarthritis (hope trial): a randomised controlled trial	Intervention
Carmona	2015	Effectiveness and cost-effectiveness of a health coaching intervention to improve the lifestyle of patients with knee osteoarthritis: cluster randomized clinical trial	Design
Coleman	2008	Effects of self-management, education and specific exercises, delivered by health professionals, in patients with osteoarthritis of the knee	Design
Crossley	2015	Exercise, education, manual-therapy and taping compared to education for patellofemoral osteoarthritis: a blinded, randomised clinical trial	Intervention
De Oliveira	2012	Impact of exercise on the functional capacity and pain of patients with knee osteoarthritis: a randomized clinical trial	Intervention
Di Giovanni	2003	Tissue-specific plantar fascia-stretching exercise enhances outcomes in patients with chronic heel pain	Intervention
Egerton	2022	Comparative effect of two educational videos on self-effcacy and kinesiophobia in people with knee osteoarthritis: an online randomised controlled trial	Intervention
Eitzen	2015	No effects of a 12-week supervised exercise therapy program on gait in patients with mild to moderate osteoarthritis: a secondary analysis of a randomized trial	Intervention
Fernandes	2010	Effcacy of patient education and supervised exercise vs patient education alone in patients with hip osteoarthritis: a single blind randomized clinical trial	Intervention
Ferrell	1997	A randomized trial of walking versus physical methods for chronic pain management	Population
Focht	2014	Group-mediated physical activity promotion and mobility in sedentary patients with knee osteoarthritis: results from the impact-pilot trial	Outcome
Focht	2017	A group-mediated physical activity intervention in older knee osteoarthritis patients: effects on social cognitive outcomes	Outcome
Gaines	2004	The effect of neuromuscular electrical stimulation on arthritis knee pain in older adults with osteoarthritis of the knee	Intervention
Guo	2021	Chinese medicine involving triple rehabilitation therapy for knee osteoarthritis in 696 outpatients: a multi-center, randomized controlled trial	Population
Hansson	2010	Effect of an education programme for patients with osteoarthritis in primary care - a randomized controlled trial	Population

First Author	Year	Title	Exclusion reasons
Holm	2020	Low-dose strength training in addition to neuromuscular exercise and education in patients with knee osteoarthritis in secondary care e a randomized controlled trial	Intervention
Holm	2021	Strength training in addition to neuromuscular exercise and education in individuals with knee osteoarthritis - the effects on pain and sensitization	Intervention
Hott	2019	Effectiveness of isolated hip exercise, knee exercise, or free physical activity for patellofemoral pain	Population)
Hott	2020	Patellofemoral pain: one year results of a randomized trial comparing hip exercise, knee exercise, or free activity	Population
Hunter	2023	Effectiveness of a new service delivery model for management of knee osteoarthritis in primary care: a cluster randomized controlled trial	Design
Huysmans	2021	Do sex and pain characteristics infuence the effectiveness of pain neuroscience education in people scheduled or total knee arthroplasty? Secondary analysis of a randomized controlled trial	Intervention
Iluch	2017	Preoperative pain neuroscience education combined with knee joint mobilization for knee osteoarthritis : a randomized controlled trial	Intervention
Jayakumar	2021	Comparison of an artificial intelligence–enabled patient decision aid vs educational material on decision quality, shared decision-making, patient experience, and functional outcomes in adults with knee osteoarthritis. A randomized clinical trial	Outcome
Jimenez	2014	Programas de educación en salud y entrenamiento de la fuerza en adultos mayores con artrosis de cadera leve a moderada	Intervention
Keefe	1990	Pain coping skills training in the management of osteoarthritic knoc pain: a comparative study	Design
Keefe	1996	Spouse-assisted coping skills training in the management of osteoarthitic knee pain	Population
Kloek	2018	Effectiveness of a blended physical therapist intervention in people with hip osteoarthritis, knee osteoarthritis, or both: a cluster randomized controlled trial	Design
Kovar	1992	Supervised fitness walking in patients with osteoarthritis of the knee	Population
Lawford	2018	Moderators of effects of internet-delivered exercise and pain coping skills training for people with knee osteoarthritis: exploratory analysis of the impact randomized controlled trial	Outcome
Li	2020	Effects of a 12-week multifaceted wearable-based program for people with knee osteoarthritis: randomized controlled trial	Outcome
Liu	2023	Efficacy and safety of tuina (chinese therapeutic massage) for knee osteoarthritis: a randomized, controlled, and crossover design clinical trial	Intervention
Lopez-Olivo	2021	A randomized controlled trial comparing two self administered educational strategies for patients with knee osteoarthritis	Outcome
Naruseviciute	2020	The effect of high-intensity versus low-level laser therapy in the management of plantar fasciitis: randomized participant blind controlled trial	Intervention

First Author	Year	Title	Exclusion reasons
Nelligan	2021	Moderators of the effect of a self-directed digitally delivered exercise program for people with knee osteoarthritis: exploratory analysis of a randomized controlled trial	Outcome
Osborne	2006	Can a disease-specific education program augment self-management skills and improve health-related quality of life in people with hip or knee osteoarthritis?	Design
Palmer	2014	Transcutaneous electrical nerve stimulation as an adjunct to education and exercise for knee osteoarthritis: a randomised controlled trial	Intervention
Robbins	2021	Is a stepped-care intervention effective in overweight and obese people with medial tibiofem-oral osteoarthritis? The streamline study: a randomised controlled trial	Design
Schmid	2013	The influence of tai chi exercise on proprioception in patients with knee osteoarthritis: results from a pilot randomized controlled trial	Outcome
Skou	2020	Cost-effectiveness of 12 weeks of supervised treatment compared to written advice in pa-tients with knee osteoarthritis: a secondary analysis of the 2-year outcome from a randomized trial	Outcome
Squiers	2020	A poster summarizing the American academy of orthopaedic surgeons knee osteoarthritis clinical practice guideline is a powerful tool for patient education: a randomized controlled trial	Outcome
Stener-Victorin	2004	Comparison between electro-acupuncture and hydrotherapy, both in combination with pa-tient education and patient education alone, on the symptomatic treatment of osteoarthritis of the hip	Intervention
Svege	2013	Exercise therapy may postpone total hip replacement surgery in patients with hip osteoar-thritis: a long-term follow-up of a randomised trial	Intervention
Svege	2015	Long-term effect of exercise therapy and patient education on impairments and activity limi-tations in people with hip osteoarthritis: secondary outcome analysis of a randomized clinical trial	Intervention
Talbot	2003	A home-based protocol of electrical muscle stimulation for quadriceps muscle strength in older adults with osteoarthritis of the knee	Intervention
Terradas-Monllor	2023	A feasibility study of home-based preoperative multimodal physiotherapy for patients sched-uled for a total knee arthroplasty who catastrophize about their pain	Intervention
Thiengwittaya-porn	2021	Development of a mobile application to improve exercise accuracy and quality of life in knee osteoarthritis patients: a randomized controlled trial	Intervention
Tsai	2016	A supplemental report to a randomized cluster trial of a 20-week sun-style tai chi for osteoar-thritic knee pain in elders with cognitive impairment	Population
Tumturk	2023	Effect of telerehabilitation-based exercise and education on pain, function, strength, proprio-ception, and psychosocial parameters in patients with knee osteoarthritis	Intervention
Van Baar	1998	The effectiveness of exercise therapy in patients with osteoarthritis of the hip or knee: a ran-domized clinical trial	Intervention

First Author	Year	Title	Exclusion reasons
Veenhof	2006	Effectiveness of behavioral graded activity in patients with osteoarthritis of the hip and/or knee: a randomized clinical trial	Design
Walsh	2020	Facilitating activity and self-management for people with arthritic knee, hip or lower back pain (fasa): a cluster randomised controlled trial	Population
Williamson	2017	An online exercise program plus automated coping skills training improved pain and function in chronic knee pain	Design
Zhou	2023	Modulation effects of different treatments on periaqueductal gray resting state functional connectivity in knee osteoarthritis knee pain patients	Intervention

Table S4. Synthesis of TPE interventions.

First Author	Year	Educational Intervention	Materials used	Provider (Specific Training)	Delivery mode	Sessions (length and frequency)	Topic of education								Combined Interventions
							SP	SM	PA	LM	PE	Diet	Stress	Sleep	
Ackerman [81]	2012	E) Arthritis SM Program	Arthritis self-help book	HEP and peer (with ST)	F2F, G (NR)	6 (2.5h, 1xW)			X		X				-
Ahmad [75]	2023	E) Education + Home-based exercise	HBE booklet	R (NR)	F2F, G (NR)	1 (30m)			X			X			Home - Based Exercise
Allen [44]	2010	E) OA SM and CBT	Written and audio materials	Health Educator (NR)	Phone calls, I	12 (NR, 1xM)		X							-
Allen [34]	2021	E) STEP-KOA	Web materials	HEP (with ST)	Web based, I + phone call, I + F2F, I	6 phone call (NR, 2xM)) + 3-7 PT: 1 (60m), others (30m)			X						Exercise + PT
Bagheri [51]	2021	E) Mindfulness + exercise	Training manuals, CDs instructions for practice	Pt, R, and SP (NR)	F2F, G (7-8)	8 (NR, 1xW)							X		Exercise
Bandak [68]	2022	E) Exercise + Education	GLAD education material	Pt (with ST)	F2F, G (Max 12)	2 (1.5h, 1xW)	X	X	X						Exercise
Bennell [73]	2014	E) PT (manual therapy, exercise and education)	NR	8 Pt (with ST)	F2F, I	10 over 12 Ws: 1-2 (45-60m), 3-10 (30m)	X		X		X				-
Bennell [32]	2015	E) Pain Coping Skills Training + Exercise	Handouts, Specific logbook, CDs	Pt (with ST)	F2F, I	10 (45m) over 12 Ws					X				Exercise
		C1) Pain Coping Skills Training	Handouts, Specific logbook, CDs	Pt (with ST)	F2F, I	10 (45m) over 12 Ws					X				-

Bennell [74]	2017	E) Coaching + PT	Information booklet	3 Nurses (with ST)	Phone call, I	6: W2 (30-45m); W4, W8, W13, W21, W25 (15-30m)		X	X							PT
Bennell [53]	2017	E) Pain coping skills training + Home strength exercise program	Web educational materials	8 Pt (1D ST)	Internet based materials + Skype calls I	8 modules (35-45m, 1xW) + 7 Skype (W2, W12 45m; W3, W4, W6, W8, W10 30m)		X	X	X	X	X				Home exercise program
Bennell [54]	2022	E1) Education, Diet + Individualized exercise program	Educational booklets	3 Pt and 5 Dieticians (NR)	Web-based + Zoom consultation	12: 6 diet, 6 PT over 6M: 1 (45m), 2-6 (20m)	X	X	X			X			X	Individualized exercise program
		E2) Education + Individualized exercise program	Educational booklets	3 Pt (NR)	Web-based + Zoom consultation	6 over 6M: 1 (45m), 2-6 (20m)	X	X	X						X	Individualized exercise program
Bezalel [61]	2010	E) Education program + SM	Home diary	Pt (NR)	F2F, G (NR)	4 (1h, 1xW)	X		X	X						SM
Brosseau [80]	2012	E) Walking + CBT	Heart rate monitor	Pt (NR)	F2F, G (NR) + F2F, I	20 G (2h, 1xW) + 6 I (NR, 1xM)			X							Walking
Chaharmahali [52]	2023	E1) Motivational interview + NM exercise	-	CP (NR)	F2F, I + Phone calls, I	6: 1 (45-60m), 2-6 (10-15m, 1xW)			X		X					NM Exercise
		E2) Mindfulness +NM exercise	Video + audio + notebook	CP (NR)	F2F, NR	6: 1 (45-60m) 2-6 (15-20m, 1xW)										NM Exercise
Cheung [50]	2019	E) Self-administered acupressure education	Written acupressure protocol	Chinese Medicine practitioner (NR)	F2F, G (4-6) + phone calls	2 (90m, 1xW) + Phone calls (NR, 2xW)	X	X								-
		C) Knee health education	Written summary	Nurse (NR)	F2F, G (NR) + phone calls, I	2 (90m, 1xW) + Phone calls (NR, 2xW)		X		X						-
Cheung [65]	2020	C) Knee health education	-	Pt or Nurse (with ST)	F2F, G (4-7)	2 (2h, 1xW)		X			X					-
Coleman [47]	2012	E) OAK CBT	Printed information	Nurses, Ot, Pt (with ST)	F2F, G (12-15)	6 (2.5h, 1xW)	X	X		X						-
Da Silva [55]	2015	E) SM education + exercise	-	4 Pt students (with ST)	F2F, G (NR)	1 (90m) + 16 (15m, 2xW)		X			X					Exercise

Esculier [41]	2018	E1) Gait retraining + Education	-	Pt (NR)	F2F, I	NR		X	X	X						Gait re-training
		E2) Education + Exercise	-	Pt (NR)	F2F, I	NR		X	X	X						Exercise
Ettinger [76]	1997	C) Health education	Videotaped, preprinted educational materials	NR (with ST)	F2F, G (10-15) + phone consultation	3 (1h, 1xM) + 17 phone consultations (NR, 1-6 2xM, 7-17 1xM)	X		X							-
Foo [57]	2020	E) Education CBT + exercise	The Knee book	2 Pt and 2 Nurses (with ST)	F2F, G (8-12)			X	X		X				X	Exercise
Ganji [45]	2018	E) Educational program	-	NR	F2F, G (13-14)	6 (1.5h 2xW)										-
		C) Single 2-hour education session	-	NR	F2F, G (NR)	1 (2h)										-
Henriksen [69]	2023	E) Exercise + Education	GLAD education material	Pt (with ST)	F2F, G (Max 12)	2 (1.5h, 1xW)	X	X	X							Exercise
Hopman-Rock [70]	2000	E) SM education program + exercise	Course book	Multi-professional team (NR)	F2F, G (max 15)	6 (1h, 1xW)	X	X				X				Exercise
James [77]	2021	E) Leaflet + Individualized home exercisebagheri	Leaflet	Pt (NR)	F2F, I	1 (30m)				X						Individualized home exercise
		C) Individualized home exercise														
Jinnouchi [46]	2023	E) Brief education for SM	SM textbook	NR	F2F, I	4 (W1: 3x30m; W5: 9x20m)			X		X					-
Khachian [71]	2020	E) SM education + exercise + medical care	Pamphlet	R (NR)	F2F, G (NR)	6 sessions (60m, 1xW) + 6 phone calls (NR)			X		X	X				Medical care
Marconcin [66]	2018	E) SM education + Exercise	-	NR	F2F, G (15 max)	24 (30m, 2xW)										Exercise
Maurer [78]	1999	C) Education + Informing material	Pamphlets + Video	Multi-professional team (NR)	NR	4 (NR)	X	X				X				Information material
Mecklenburg [56]	2018	E) Hinge Health APP	Hinge Health APP	-	Trough Hinge Health APP, I	At participant will	X	X	X			X				-
Mellor [62]	2018	E) Education + exercise	Handouts + DVD	Pt (with ST)	F2F, I	14 over 8 Ws: 1 (1h), 2-14 (30m)			X	X						Exercise
Murphy [42]	2016	E1) Tailored pacing	Learning module	Ot (with ST)	F2F, I	3 (first 1h, then 30-45m) every 7-10 Ds			X	X						-

		E2) General pacing intervention	Learning module	Ot (with ST)	F2F, I	3 (first 1h, then 30-45m) every 7-10 Ds			X						-
Nagasawa [79]	2022	E) Acceptance and Commitment Therapy + exercise	Textbook based on the advice of a psychologist	3 Pt (3x2h ST)	F2F, I	8 (NR, 1xW)					X				Exercise
Nunez [83]	2006	E) Education + functional readaptation	Written information	Health Educator (with ST)	1,4 F2F, I; 2,3 F2F, G (10-12)	4 (1xW): 1, 4 (30m); 2, 3 (1.5h)		X	X	X					Exercise and Medical care
Olsen [43]	2022	E) Education + Basic Body Awareness Therapy	-	OS and Pt (NR)	F2F, G (open)	1 (3.5h) + 12 (90m, 1xW)	X	X		X					-
		C) Patient education	-	OS and Pt (NR)	F2F, G (NR)	1 (3.5h)	X	X		X					-
Park [35]	2017	C) Health education program	-	NR (2x4h ST)	F2F, G (NR)	16 (45m, 2xW)	X								-
Poulsen [33]	2013	E) Patient education + manual therapy	Papers with Recommendations for ADL + exercise	Pt (with ST)	Interviews F2F, I + F2F, G (NR)	5: 1 I (45m), 2-4 G (90m), 5 I (30m), Frequency NR	X		X		X				Manual Therapy
		C1) Patient education	Recommendations for ADL + exercise	Pt (with ST)	Interviews F2F, I + F2F, G (NR)	5: 1 I (45m), 2-4 G (90m), 5 I (30m), Frequency NR	X		X		X				-
Rabiei [64]	2023	E) PSE + Pilates Exercises	-	Pt (with ST)	F2F, I	3 (30-60 m, NR)			X		X				Pilates Exercises
Rezende [39]	2016	E) Multiprofessional education program	Booklet + video + online material	Multi-professional team (NR)	F2F, G (28-29)	2 (10h at 1, 2 or 3 M distance) + Ev. 12 Phone calls (NR, 1xM)	X	X	X			X			-
Rezende [38]	2017	E) Multiprofessional education program	Booklet + video + online material	Multi-professional team (NR)	F2F, G (28-29)	2 (10h at 1, 2 or 3 M distance) + Ev. 12 Phone calls (NR, 1xM)	X	X	X			X			-
Rezende [40]	2017	E) Multiprofessional education program	Booklet + video + online material	Multi-professional team (NR)	F2F, G (28-29)	2 (10h at 1, 2 or 3 M distance) + Ev. 12 Phone calls (NR, 1xM)	X	X	X			X			-
Rezende [72]	2021	E) Multiprofessional education program + medications	Written and video educational material	Multi-professional team (NR)	F2F, G (NR)	2 (8h, 1x2M)	X	X	X	X		X		X	Medical care
Rini [48]	2021	E) PainCOACH intervention	PainCOACH app	- (virtual coach)	Web-based PCST, I	8 (35-45m) in 8-10 Ws		X	X	X					-
Saffari [82]	2018	E) Educational CBT program	CD-ROM + Booklet	NR	F2F, G (8-10)	7 (60-90m) over 1M									-
Saw [58]	2016	E) Education + exercise	"Living with Osteoarthritis" workbook	R and 2 Pt (with ST)	F2F, G (Max 12)	6 (1h, 1xW)		X	X			X	X		Exercise

Skou [59]	2015	E) "MEDIC" intervention	DVD	Pt and dietician (with ST)	F2F, NR + phone calls, I	2 (60m) + ev. 4 Diet (60m, 1xM)	X			X		X	NM Exercise
Skou [60]	2016	E) "MEDIC" intervention	DVD	Pt and dietician (with ST)	F2F, NR + phone calls, I	2 (60m) + ev. 4 Diet (60m, 1xM)	X			X		X	NM Exercise
Song [36]	2022	C) Health education + SM	-	NR	F2F, NR	12 (1h, 1xW)		X	X				SM
Sullivan [67]	1998	E) Education + Walking	-	NR	F2F, G (10-15)	24 (NR, 3xW)			X	X			Walking
Taglietti [37]	2018	C) Education group	NR	Multi-professional team (NR)	F2F, G (5)	8 (2h, 1xW)	X		X	X	X		-
Victor [49]	2005	E) Education program	Booklet	2 Nurses (NR)	F2F, G (6-8)	1 home visit + 4 (1h, NR)	X	X	X				-
Wilson [63]	2023	E) Education + exercise	Handouts + DVD	Pt (with ST)	F2F, I	14 over 8 Ws: 1 (1h), 2-14 (30m)			X	X			Exercise

Legend of acronyms. C (Control group); CP (Clinical Psychologist); D (Day); E (Experimental group); Ev (Eventually); F2F (Face-to-Face); G (Group); h (hour); HEP (Health Education Personnel); I (Individually); LM (Load Management); m (minute); M (Month); NM (Neuro-Muscular); NR (Not Reported); OS (Orthopaedic Surgeon); Ot (Occupational therapist); PA (Physical Activity); PE (Pain Education); PT (Physical Therapy); Pt (Physiotherapist); R (Researcher); SM (Self-Management); SP (Specific Pathology); SP (Sport Psychologist); W (Week).

Table S5. Risk of Bias assessment according to the revised JBI critical appraisal tool for RCTs.

First Author	Year	Item1	Item 2	Item 3	Item 4	Item 5	Item 6	Item 7	Item 8	Item 9	Item 10	Item 11	Item 12	Item 13	% of Yes
Ackerman [81]	2012	🟢	🟢	🟢	🔴	🔴	🟢	🔴	🟢	🟢	🟢	🟢	🟢	🔴	69.23%
Ahmad [75]	2023	🟢	🟢	🟢	🔴	🟡	🔴	🟢	🟢	🟢	🟢	🟢	🟢	🟢	76.92%
Allen [44]	2010	🟢	🔴	🔴	🔴	🟡	🟢	🟡	🟢	🔴	🟢	🟢	🟢	🟢	53.85%
Allen [34]	2021	🟢	🟢	🟢	🔴	🔴	🟢	🟢	🟢	🟢	🟢	🟢	🟢	🟡	76.92%
Bagheri [51]	2021	🟢	🟢	🟢	🟡	🔴	🟢	🟢	🟢	🟢	🟢	🟢	🟡	🟢	76.92%
Bandak [68]	2022	🟢	🟢	🟢	🔴	🔴	🟡	🟢	🟢	🟢	🟡	🟢	🟢	🟢	69.23%
Bennell [73]	2014	🟢	🟢	🟢	🟢	🔴	🟡	🟢	🟢	🟢	🟢	🟢	🟡	🔴	69.23%
Bennell [32]	2015	🟢	🟢	🟢	🟡	🔴	🟢	🟢	🟢	🟢	🟢	🟢	🟢	🟢	84.62%
Bennell [74]	2017	🟢	🟢	🟢	🟡	🔴	🟢	🔴	🟢	🟢	🟢	🟢	🟢	🟢	76.92%
Bennell [53]	2017	🟢	🟢	🟢	🔴	🔴	🟢	🔴	🟢	🟢	🟢	🟢	🟢	🟢	76.92%
Bennell [54]	2022	🟢	🟢	🟢	🟡	🔴	🟢	🟢	🟢	🟢	🟡	🟢	🟢	🟢	76.92%
Bezalel [61]	2010	🟢	🟢	🟢	🔴	🟡	🟢	🟢	🟢	🟢	🟢	🟢	🟡	🟢	76.92%
Brosseau [80]	2012	🟢	🟡	🟢	🔴	🔴	🟢	🟢	🟢	🟢	🟢	🟢	🟡	🟡	61.54%

Chaharmahali [52]	2023	🟢	🟢	🟢	🔴	🔴	🟢	🟢	🟢	🟢	🔴	🟢	🟡	🟢	69.23%
Cheung [50]	2019	🟢	🟢	🟢	🔴	🔴	🟢	🟢	🟢	🟢	🔴	🟢	🔴	🟢	69.23%
Cheung [65]	2020	🟢	🟡	🔴	🔴	🔴	🟢	🟢	🟢	🟢	🔴	🟢	🔴	🔴	46.15%
Coleman [47]	2012	🟢	🟢	🟢	🔴	🟡	🟢	🟢	🟢	🟢	🟢	🟢	🟢	🟢	84.62%
Da Silva [55]	2015	🟢	🟢	🟢	🔴	🔴	🟢	🟢	🟢	🟡	🟢	🟢	🟢	🟢	76.92%
Esculier [41]	2018	🟢	🟢	🟢	🔴	🔴	🟢	🟢	🟢	🟢	🔴	🟢	🟡	🟢	69.23%
Ettinger [76]	1997	🟢	🟢	🟢	🔴	🟢	🟡	🟡	🟢	🟡	🟢	🟢	🟢	🟢	69.23%
Foo [57]	2020	🟢	🔴	🟢	🔴	🔴	🟢	🟢	🟢	🟢	🟢	🟢	🟢	🟢	76.92%
Ganji [45]	2018	🟢	🟡	🟡	🔴	🔴	🟢	🟢	🟢	🟢	🟢	🟡	🟡	🟢	53.85%
Henriksen [69]	2023	🟢	🟢	🟢	🔴	🔴	🟡	🟢	🟢	🟢	🔴	🟢	🔴	🟢	61.54%
Hopman-Rock [70]	2000	🔴	🟡	🟡	🔴	🔴	🟡	🟡	🟢	🟢	🟡	🟢	🟢	🔴	30.77%
James [77]	2021	🟢	🟡	🟢	🔴	🔴	🟢	🔴	🟢	🟢	🔴	🟢	🔴	🟢	53.85%
Jinnouchi [46]	2023	🟢	🟢	🟢	🔴	🔴	🟢	🔴	🟢	🟢	🔴	🟢	🟢	🟢	69.23%
Khachian [71]	2020	🟢	🟢	🟢	🔴	🔴	🟡	🟢	🟢	🟢	🟢	🟢	🟢	🟢	76.92%
Marconcin [66]	2018	🟢	🟢	🟢	🔴	🔴	🟢	🟢	🟢	🟡	🟢	🟡	🟢	🟡	61.54%
Maurer [78]	1999	🟢	🟡	🟢	🟡	🟡	🟢	🟢	🟢	🟢	🟡	🟡	🟡	🔴	46.15%
Mecklenburg [56]	2018	🟢	🟢	🟢	🔴	🔴	🟢	🔴	🟡	🟡	🟢	🟢	🟡	🟢	53.85%
Mellor [62]	2018	🟢	🟢	🟢	🔴	🟡	🔴	🟢	🟢	🟢	🟢	🟢	🟢	🟢	76.92%
Murphy [42]	2016	🟡	🟡	🟢	🔴	🔴	🟢	🟢	🟢	🟢	🟢	🟢	🟢	🟢	69.23%
Nagasawa [79]	2022	🟢	🟡	🟢	🔴	🔴	🟢	🟢	🟢	🟡	🟢	🟡	🔴	🔴	46.15%
Nunez [83]	2006	🟢	🟢	🟢	🟡	🟡	🟢	🟢	🟢	🟢	🟢	🟢	🟡	🟢	76.92%
Olsen [43]	2022	🟢	🟢	🟢	🔴	🔴	🔴	🟢	🟢	🟢	🟢	🟢	🟡	🟢	69.23%
Park [35]	2017	🟢	🟡	🟢	🔴	🔴	🟢	🟢	🟢	🟡	🟢	🟢	🟡	🟢	61.54%
Poulsen [33]	2013	🟢	🟢	🟢	🔴	🔴	🟢	🟢	🟢	🟢	🟢	🟢	🟢	🟢	84.62%
Rabiei [64]	2023	🟢	🟢	🟢	🔴	🟡	🟢	🟢	🟢	🟢	🟢	🟢	🟢	🟢	84.62%
Rezende [39]	2016	🟢	🟡	🟢	🔴	🔴	🟡	🟢	🟢	🟡	🟢	🟡	🟢	🟢	53.85%
Rezende [38]	2017	🟢	🟡	🟢	🔴	🔴	🟡	🟢	🟢	🟡	🟢	🟡	🟡	🟢	46.15%
Rezende [40]	2017	🟢	🟡	🟢	🔴	🔴	🟡	🟢	🟢	🟡	🟢	🟡	🟢	🟢	53.85%
Rezende [72]	2021	🟡	🟢	🟢	🔴	🔴	🟡	🟢	🟢	🟡	🔴	🔴	🟡	🟢	38.46%

Rini [48]	2021	Yes	Yes	Yes	NO	NO	Yes	Yes	Unclear	Yes	Yes	Yes	Unclear	Yes	69.23%
Saffari [82]	2018	Unclear	NO	Yes	NO	Unclear	Yes	Unclear	Yes	Yes	Yes	Yes	Yes	Yes	61.54%
Saw [58]	2016	NO	Unclear	Yes	NO	NO	Unclear	Yes	Unclear	Unclear	Yes	Yes	Yes	Unclear	38.46%
Skou [59]	2015	Yes	Yes	Yes	NO	NO	Yes	Yes	Yes	Yes	Yes	Yes	Yes	Yes	84.62%
Skou [60]	2016	Yes	Yes	Yes	NO	NO	Yes	Yes	Yes	Yes	Yes	Yes	Yes	Yes	84.62%
Song [36]	2022	Yes	Yes	Yes	NO	NO	Yes	Yes	Yes	Yes	Yes	Yes	Unclear	Yes	76.92%
Sullivan [67]	1998	Unclear	Unclear	Yes	NO	Unclear	Unclear	Yes	Unclear	Unclear	Unclear	Yes	Unclear	Unclear	23.08%
Taglietti [37]	2018	Yes	Yes	Yes	NO	NO	Yes	Yes	Yes	Yes	Yes	Yes	Unclear	Yes	76.92%
Victor [49]	2005	Unclear	Yes	Unclear	Unclear	Unclear	Unclear	Yes	Yes	Yes	Yes	Yes	Unclear	Yes	53.85%
Wilson [63]	2023	Yes	Yes	Yes	NO	Unclear	NO	Yes	Yes	Yes	Yes	Yes	Yes	Yes	76.92%

Legend of colors. ● = Yes; ● = Unclear; ● =NO. The references are reported in square brackets.

Legend of acronyms. JBI (Johanna Briggs Institute); RCTs (Randomized Controlled Trials)

Table S6. Results on included studies on pain.

FIRST AUTHOR	YEAR	POPULATION (OSTEOARTHRITIS)	INTERVENTION	COMPARISON	RESULTS (PAIN)
Allen [44]	2010	Hip OA, Knee OA (n=515)	TPE: OA self-management cognitive behavioral therapy	C1: Health education<br>C2: Usual care	Greater pain reduction at 12 months in TPE group compared to C1 (-0.6; 95%CI -1, -0.2) and C2 (-0.4; 95% CI -0.8, 0.1) groups
Hopman-Rock [70]	2000	Hip OA, Knee OA (n=105)	TPE: Self-management program	Waitlist	No significant difference at 6 months follow-up
Murphy [42]	2016	Hip OA, Knee OA (n=193)	TPE1: Tailored pacing<br>TPE2: General pacing intervention	Usual care	No significant differences among groups at 10 weeks and 6 months
Park [35]	2017	Hip OA, Knee OA (n=112)	TPE: Health education program (HEP)	Chair yoga exercise (CY)	Significantly greater pain decrease for the yoga group over 8 weeks (CY β = −1.0, HEP β = −0.4, p = .048)
Rini [48]	2021	Hip OA, Knee OA (n=113)	TPE: Web-based Pain Coping Skills Training	Assessment	No significant difference at 5 and 10 weeks
Saw [58]	2016	Hip OA, Knee OA (n=74)	TPE: Education + exercise	Usual care	Greater reduction in the TPE group for pain perception at 6 weeks: 2.44 (CI95% 0.6, 4.3); at 12 weeks: 2.05 (CI95% 0.51, 3.6) and at 6 months: 2.24(CI95% 0.55, 3.9). Greater reduction in the TPE group for pain interference at 6 weeks: 2.95(CI95% 0.73, 5.2); at 12 weeks: 2.03(CI95% 0.5, 3.6); and at 6 months 2.69(CI95% 0.66, 4.7)

FIRST AUTHOR	YEAR	POPULATION (OSTEOARTHRITIS)	INTERVENTION	COMPARISON	RESULTS (PAIN)
Bennell [73]	2014	Hip OA (n=102)	TPE: Education + physiotherapy	Sham (inactive ultrasound)	No significant differences at 13 and 36 weeks (similar reduction of 10-30mm)
Olsen [43]	2022	Hip OA (n=101)	TPE: Patient education + Basic body awareness therapy	Patient education	No significant improvement in neither group at post-treatment
Poulsen [33]	2013	Hip OA (n=118)	TPE1: Education<br><br>TPE2: Education + manual therapy	Education pamphlet (MCI)	TPE2 group: greater reduction compared to MCI at 6 week (-1.9; 95%CI -2.9, -0.9) and at 12 months (per-protocol analysis: -1.1; 95%CI -2.1, -0.1)
Allen [34]	2021	Knee OA (n=345)	TPE: STepped Exercise Program for patients with Knee OsteoArthritis (STEP-KOA)	Arthritis education	Limited effects at 9 months in favor of STEP-KOA group:-1.4 (95% CI -2.3, -0.6)
Bandak [68]	2022	Knee OA (n=206)	TPE: Education + exercise	Intra-articular saline injection	TPE group: better but not statistically significant results compared to control group at week 9 (MD 2.7, 95%CI -0.6, 6)
Bennell [32]	2016	Knee OA (n=222)	TPE1: Pain coping skills training<br><br>TPE2: Pain coping skills training + exercise	Exercise	No significant differences among groups in pain at 12 and 52 weeks. TPE2: greater improvement at 32 weeks compared to Exercise group (Mean difference 9.4, CI95% 1.0, 17.9) and TPE1 group (Mean difference 8.4, CI95% 0.3, 16.6)
Bennell [74]	2017	Knee OA (n=168)	TPE: Coaching + physical therapy	Physical therapy	No significant difference at 6, 12 and 18 months
Bennell [54]	2022	Knee OA (n=415)	TPE1: Education + exercise<br><br>TPE2: Education + diet + exercise	Educational material consultation	Greater improvement in TPE2 group at 6 months compared to TPE1 group (-0.6; 95%CI -1.1, -0.2) and compared to material consultation (-1.5; 95%CI -2.1, -0.8). TPE1 group: better results than material consultation (-0.8; 95%CI -1.5, -0.2). Similar reduction is shown at 12 months follow-up
Bezalel [61]	2010	Knee OA (n=50)	TPE: Group education + self-management	Brief course in short-wave diathermy treatment	Minimal difference at 12 weeks in favor of T group (−2.0; 95%CI −3.7, −0.3); no difference at 4 weeks
Chaharmahali [52]	2023	Knee OA (n=60)	TPE1: Motivational interview+ exercises<br><br>TPE2: Mindfulness + exercise	Exercise	TPE1 group: better improvement post-treatment than Exercise group (95% CI 1.33-2.55). TPE2 group: no difference with Exercise group

FIRST AUTHOR	YEAR	POPULATION (OSTEOARTHRITIS)	INTERVENTION	COMPARISON	RESULTS (PAIN)
Cheung [50]	2019	Knee OA (n=35)	TPE: Knee health education	Self-administered acupressure	No significant differences post-treatment
Cheung [65]	2020	Knee OA (n=38)	TPE: Knee health education	Electromoxibustion	Minimal similar effects in both groups at post-treatment
Coleman [47]	2012	Knee OA (n=147)	TPE: Cognitive behavioral therapy	Usual care	Minimal differences at 8 weeks in favor of TPE group (-1.46; 95% CI -2.18, -0.73) but not maintained at 6 months
Da Silva [55]	2015	Knee OA (n=41)	TPE: Self-management + exercise	Minimal education	Greater reduction in pain for TPE group at 8 weeks (IG 2.60 ± 1.55; CG 4.00 ± 1.56)
Ettinger [76]	1997	Knee OA (n=439)	TPE: Health education	C1: Aerobic exercise<br>C2: Resistance exercise	C1: 12% lower pain score (2.1 +/- 0.05 vs 2.4 +/- 0.05 units)<br>C2: 8% lower pain score (2.2 +/- 0.06 vs 2.4 +/- 0.05 units)
Foo [57]	2020	Knee OA (n=300)	TPE: Educational cognitive behavioral therapy + exercise	Usual care	TPE group: greater pain reduction compared to Usual care group with increased mean difference over time. Post-intervention: CG −0.190 (95%CI −1.108, 0.728), TPE -3.067 (95%CI 2.159, 3.974); 1 months follow-up CG -0.432 (95%CI −1.901, 2.765), TPE -4.264 (95%CI 1.958, 6.570); 6 months follow-up: CG -0.603 (95%CI −1.732, 2.938), TPE -8.926 (95%CI 6.618, 11.234)
Ganji [45]	2018	Knee OA (n=82)	TPE: Educational group program	Minimal education	Significant differences at 8 weeks in favor of TPE group (-1.32, p=0.009)
Henriksen [69]	2023	Knee OA (n=206)	TPE: Education + exercise	Intra-articular saline injection	No significant difference at 1 year follow-up
Khachian [71]	2020	Knee OA (n=80)	TPE: Self-management + exercise + standard medical care	Standard medical care	TPE group: greater improvement post-treatment (+10.26, p<0,001)
Marconcin [66]	2018	Knee OA (n=80)	TPE: Group self-management + exercise	Educational group	No significant difference at post-treatment: SMEG −16.0 (17.8); EC −6.0 (16.2)
Rabiei [64]	2023	Knee OA (n=54)	TPE: Pain neuroscience education + Pilates exercise	Pilates exercise	No significant difference at post-treatment (pain reduced by 31.1% in TPE group and 24.3% in Pilates group)
Rezende [39]	2016	Knee OA (n=228)	TPE: Multiprofessional education program	Educational material delivery	No significant difference at 4 and 12 months
Rezende [38]	2017	Knee OA (n=228)	TPE: Multiprofessional education program	Educational material delivery	No significant difference at 1 and 2 years

FIRST AUTHOR	YEAR	POPULATION (OSTEOARTHRITIS)	INTERVENTION	COMPARISON	RESULTS (PAIN)
Rezende [72]	2021	Knee OA (n=222)	TPE: Multiprofessional education program + medication	Medication	TPE group: greater pain reduction at 6 months (mean difference 1.41, 95% CI 0.07, 2.76) and at 12 months (mean difference 2.09, 95% CI 0.75, 3.42). No difference at 24 months
Skou [59]	2015	Knee OA (n=100)	TPE: MEDIC treatment (education, exercise, insoles, weight loss and/or pain medication)	Usual care	Greater reduction in TPE group of -9.0 (CI95% -15.0, -3.0) at 1 year
Skou [60]	2016	Knee OA (n=100)	TPE: MEDIC treatment (education, exercise, insoles, weight loss and/or pain medication)	Usual care	Greater reduction in the TPE group in peak pain intensity of 15.4 (95%CI 2.6, 28.2), and in pain after 30 min of walking of 32.6 (95%CI 18.1-45.0) at 3 months
Song [36]	2022	Knee OA (n=42)	TPE: Health education + self-management program	Tai Chi	WOMAC pain subscale −5.09 (95% CI −7.74,−2.43) post treatment and −2.97 (95% CI −5.16,−0.78) at 3 months in favor of Tai Chi group; no statistically significant differences at 6 months
Sullivan [67]	1998	Knee OA (n=102)	TPE: Education + walking	Usual care	No significant difference at 1 months and 1 year
Taglietti [37]	2018	Knee OA (n=60)	TPE: Education group	Aquatic exercise	No significant difference between groups in VAS scale.<br>Greater improvement in WOMAC pain subscale in aquatic exercise group compared to TPE group (MD –3.8; 95% CI –8.7, –1), not maintained at 3 months (MD -3.2; 95%CI -8, 1.6)
Victor [49]	2005	Knee OA (n=193)	TPE: Educational program	Waitlist	No significant difference at 1 months and 1 year
FIRST AUTHOR	YEAR	POPULATION (KNEE PAIN)	INTERVENTION	COMPARISON	RESULTS (PAIN)
Bennell [53]	2017	Chronic Knee Pain (n=148)	TPE: Pain coping skills training + exercise	Online educational materials	TPE group: greater pain reduction at 3 months (mean difference 1.6; 95%CI 0.9, 2.3) and 9 months (mean difference 1.1; 95%CI 0.4, 1.8)
Jinnouchi [46]	2023	Chronic Knee Pain (n=46)	TPE: Brief education for self-management	Written educational material	Nearly statistically significant pain reduction in favor of TPE group: −1.5 (95%

FIRST AUTHOR	YEAR	POPULATION (OSTEOARTHRITIS)	INTERVENTION	COMPARISON	RESULTS (PAIN)
					CI −3.2, 0.2) at 12 weeks. No significant difference at 4 weeks
Mecklenburg [56]	2018	Chronic Knee Pain (n=155)	TPE: Hinge Health Digital Care Program 12-week	3 digital pieces of education, part of the Hinge Health Digital Care Program	TPE group: greater pain reduction compared to control group of –7.7 (95%CI –12.3, –3) at 12 weeks
Bagheri [51]	2021	Patello-femoral pain (n=30)	TPE: Mindfulness + exercise	Exercise	TPE group: greater pain reduction at post treatment (-4.2; 95% CI -8.1, -0.3) and 2 months (-6.5; 95% CI -10.9, -2.1)
Esculier [41]	2018	Patello-femoral pain (n=69)	TPE1: Education + gait retraining<br><br>TPE2: Education + exercise	Education	No differences among the 3 groups (similar improvement of 0-2 at 4 weeks, 0-2 at 8 weeks and 0-3 at 20 weeks)

FIRST AUTHOR	YEAR	POPULATION (TENDINOPATHY)	INTERVENTION	COMPARISON	RESULTS (PAIN)
Mellor [62]	2018	Gluteal tendinopathy (n=204)	TPE: Education + exercise	C1: Corticosteroid injection<br><br>C2: Wait and see	At 8 weeks: greater pain reduction in TPE and C1 than in C2 groups (respectively −2.2 (CI95% −2.89, −1.54); −1.17 (CI95% −1.85, −0.50)); greater pain reduction in TPE than in C1 (−1.04 (CI95% −1.72, −0.37)). At 52 weeks: no difference between TPE and C1 groups; both these groups reported greater pain reduction than C2 group (respectively −1.13 (−1.93 to −0.33) and −0.87 (−1.68 to −0.07)
Wilson [63]	2023	Gluteal tendinopathy (n=204)	TPE: Education + exercise	C1: Corticosteroid injection<br><br>C2: Wait and see	Greater reduction in the TPE group in peak pain intensity of 15.4 (95%CI 2.6, 28.2), and in pain after 30 min of walking of 32.6 (95%CI 18.1-45.0) at 3 months than C1

Legend of acronyms. CG (Control Group); CI (Confidence Interval); CY (Chair Yoga); HEP (Health Education Program), MCI (Education Pamphlet); OA (Osteoarthritis); STEP-KOA (STepped Exercise Program for patients with Knee OsteoArthritis); TPE (Therapeutic patient Education); VAS (Visual Analogue Scale); WOMAC (Western Ontario and McMaster University Arthritis Index).

Table S7. Results of included studies on function.

FIRST AUTHOR	YEAR	POPULATION (OSTEOARTHRITIS)	INTERVENTION	COMPARISON	RESULTS (FUNCTION)
Bennell [73]	2014	Hip OA (n=102)	TPE: Education + physiotherapy	Sham (inactive ultrasound)	No significant differences between groups in WOMAC physical function subscale at 13 and 36 weeks (similar reduction of 0-10 points)
Olsen [43]	2022	Hip OA (n=101)	TPE: Patient education + Basic body awareness therapy	Patient education	No significant improvement in neither group at HOOS 2 post-treatment
Allen [34]	2021	Knee OA (n=345)	TPE: STepped Exercise Program for patients with Knee OsteoArthritis (STEP-KOA)	Arthritis education	Limited effects at 9 months in favor of TPE group, WOMAC function subscale −4.6 (95% CI −7.4, −1.9)
Bennell [32]	2016	Knee OA (n=222)	TPE1: Pain coping skills training<br><br>TPE2: Pain coping skills training + exercise	Exercise	Greater improvement in WOMAC function scale in TPE2 compared to Exercise group at 12 and 32 weeks (Mean difference 3.7, CI95% 0.4, 7.0; and mean difference 4.4, CI95% 0.2, 8.7, respectively), not maintained at 52 weeks and compared to TPE1 group at 12, 32 and 52 weeks (Mean difference 7.9, CI95% 4.7, 11.2; Mean difference 6.6, CI95% 2.3, 10.8; and mean difference 5.5, CI95% 1.6, 9.3, respectively)
Bennell [74]	2017	Knee OA (n=168)	TPE: Coaching + physical therapy	Physical therapy	No significant difference in WOMAC physical function subscale at 6, 12 and 18 months
Bennell [54]	2022	Knee OA (n=415)	TPE1: Education + exercise<br><br>TPE2: Education + diet + exercise	Educational material consultation	Greater improvement in WOMAC physical function subscale in TPE2 group at 6 months compared to TPE1 group (-2.8; 95%CI -4.7, -0.8) and compared to control group (- 9.8; 95%CI -12.5, -7.0). TPE1 showed better results than control group (-7.0; 95%CI -9.7, -4.2). Similar reduction was shown at 12 months follow-up
Bezalel [61]	2010	Knee OA (n=50)	TPE: Group education + self-management	Brief course in short-wave diathermy treatment	Minimal differences in WOMAC physical function subscale at 12 weeks in favor of TPE group (−5.9; 95%CI −10.1, −1.7); no difference at 4 weeks
Chaharmahali [52]	2023	Knee OA (n=60)	TPE1: Motivational interview+ exercises<br><br>TPE2: Mindfulness + exercise	Exercise	TPE1 showed better improvement post-treatment at WOMAC scale than TPE2 (95% CI 0.70-4.61); no difference between TPE2 and Exercise groups

FIRST AUTHOR	YEAR	POPULATION (OSTEOARTHRITIS)	INTERVENTION	COMPARISON	RESULTS (FUNCTION)
Coleman [47]	2012	Knee OA (n=147)	TPE: Cognitive behavioral therapy	Usual care	Significant differences in WOMAC physical function subscale in favor of TPE group at 8 weeks (-5.55; 95% CI -7.38, -3.31) and at 6 months (-4.35; 95% CI -6.20, -0.91)
Da Silva [55]	2015	Knee OA (n=41)	TPE: Self-management + exercise	Minimal education	Greater improvement at Lequesne Index in the TPE group at 8 weeks (IG: 2.30 ± 1.36; CG 3.13 ± 1.45)
Ettinger [76]	1997	Knee OA (n=439)	TPE: Health education	C1: Aerobic exercise<br>C2: Resistance exercise	C1 group: better (mean [+/- SE]) on the 6-minute walk test (1507 +/- 16 vs 1349 +/- 16 ft; P<.001), mean (+/-SE) time to climb and descend stairs (12.7 +/- 0.4 vs 13.9 +/- 0.4 seconds; P=.05), time to lift and carry 10 pounds (9.1 +/- 0.2 vs 10.0 +/- 0.1 seconds; P<.001), and mean (+/-SE) time to get in and out of a car (8.7 +/- 0.3 vs 10.6 +/- 0.3 seconds; P<.001)<br>C2 group: greater distance on the 6-minute walk (1406 +/- 17 vs 1349 +/- 16 ft; P=.02), faster times on the lifting and carrying task (9.3 +/- 0.1 vs 10.0 +/- 0.16 seconds; P=.001), and the car task (9.0 +/- 0.3 vs 10.6 +/- 0.3 seconds; P=.003)
Khachian [71]	2020	Knee OA (n=80)	TPE: Self-management + exercise + standard medical care	Standard medical care	Greater improvement for TPE in KOOS ADL (+11.43, p<0,001) and KOOS sport and recreational activity post-treatment (15.75, p<0,001)
Marconcin [66]	2018	Knee OA (n=80)	TPE: Group self-management + exercise	Educational group	No significant difference in KOOS ADL at post-treatment: SMEG −16.0 (16.7); EC −8.7 (13.6)
Rabiei [64]	2023	Knee OA (n=54)	TPE: Pain neuroscience education + Pilates exercise	Pilates exercise	No significant difference in WOMAC physical function subscale post-treatment (limitation reduced by 21.7% in TPE group and 18.9% in Pilates group)
Rezende [39]	2016	Knee OA (n=228)	TPE: Multiprofessional education program	Educational material delivery	No significant difference between groups at WOMAC scale at 4 and 12 months
Rezende [38]	2017	Knee OA (n=228)	TPE: Multiprofessional education program	Educational material delivery	No significant differences between group at WOMAC, TUG and FTSTS at 1 and 2 years
Rezende [40]	2017	Knee OA (n=228)	TPE: Multiprofessional education program	Educational material delivery	No significant differences between group at TUG and FTSTS at 1 year

FIRST AUTHOR	YEAR	POPULATION (OSTEOARTHRITIS)	INTERVENTION	COMPARISON	RESULTS (FUNCTION)
Rezende [72]	2021	Knee OA (n=222)	TPE: Multiprofessional education program + medication	Medication	Greater improvement in WOMAC function in TPE at 6 months (mean difference 4.12, 95% CI 1.55, 6.69), at 12 months (mean difference 7.27, 95% CI 4.73, 9.81), and at 24 months (mean difference 3.87, 95% CI 1.19, 6.54)
Skou [59]	2015	Knee OA (n=100)	TPE: MEDIC treatment (education, exercise, insoles, weight loss and/or pain medication)	Usual care	Greater reduction in KOOS ADL subscale in TPE group compared to the UC group of -11.2 (CI95% -17.1, -5.4) at 1 year
Song [36]	2022	Knee OA (n=40)	TPE: Health education + self-management program	Tai Chi	WOMAC function subscale −11.21 (95% CI −15.46,−6.97) post treatment, −7.32 (95% CI −12.18,−2.45) at 3 months and −5.16 (95% CI −10.12,−0.20) at 6 months in favor of Tai Chi group
Sullivan [67]	1998	Knee OA (n=102)	TPE: Education + walking	Usual care	Minimal difference in AIMS physical activity subscale at 1 month in favor of TPE group, not maintained at 1 year follow-up

FIRST AUTHOR	YEAR	POPULATION (KNEE PAIN)	INTERVENTION	COMPARISON	RESULTS (FUNCTION)
Bennell [53]	2017	Chronic Knee Pain (n=148)	TPE: Pain coping skills training + exercise	Online educational materials	Greater WOMAC physical function improvement in the TPE group at 3 months (mean difference 9.3; 95%CI 5.9, 12.7) and at 9 months (mean difference 7.0; 95%CI 3.4, 10.5)
Jinnouchi [46]	2023	Chronic Knee Pain (n=46)	TPE: Brief education for self-management	Written educational material	Statistically significant better in favor of TPE group at 12 weeks: KOOS4 9.4 (95% CI 2.3, 16.4). No significant differences at 4 weeks
Mecklenburg [56]	2018	Chronic Knee Pain (n=155)	TPE: Hinge Health Digital Care Program 12-week	3 digital pieces of education, part of the Hinge Health Digital Care Program	TPE group showed greater pain reduction on KOOS physical function subscale compared to control group of –7.2 (95%CI –11.5, –3) at 12 weeks
Bagheri [51]	2021	Patello-femoral pain (n=30)	TPE: Mindfulness + exercise	Exercise	Greater increase in Knee function (KOS ADL and KOS Sport) in TPE group at post intervention (8.4; 95% CI 3.4, 13.4) and at 2 months (13; 95% CI 7.7, 18.3)

FIRST AUTHOR	YEAR	POPULATION (OSTEOARTHRITIS)	INTERVENTION	COMPARISON	RESULTS (FUNCTION)
Esculier [41]	2018	Patello-femoral pain (n=69)	TPE1: Education + gait retraining<br><br>TPE2: Education + exercise	Education	No differences in KOOS ADL improvement among the 3 groups (similar improvement of 11-14 points at 4 weeks, 14-18 at 8 weeks and 15-20 at 20 weeks)

Legend of acronyms. CG (Control Group); CI (Confidence Interval); CY (Chair Yoga); FTSTS (Five Times Sit To Stand Test); HEP (Health Education Program), HOOS (Hip disability and Osteoarthritis Outcome Score); KOOS (Knee Injury and Osteoarthritis Outcome Score); MCI (Education Pamphlet); OA (Osteoarthritis); STEP-KOA (STepped Exercise Program for patients with Knee OsteoArthritis); TPE (Therapeutic Patient Education); TUG (Time Up and Go); VAS (Visual Analogue Scale); WOMAC (Western Ontario and McMaster University Arthritis Index).

Table S8. Results of included studies on disability.

FIRST AUTHOR	YEAR	POPULATION (OSTEOARTHRITIS)	INTERVENTION	COMPARISON	RESULTS (DISABILITY)
Ahmad [75]	2023	Knee OA (n=80)	TPE: Education + exercise	Usual physiotherapy	Greater disability reduction in TPE group (mean difference in of 16.3 (CI 95% 14.8 to 17.8, p<0.001) than in the control group (mean difference of 2.3 (CI 95% 1.7 to 2.9, p<0.001)) at 8 weeks
Allen [34]	2021	Knee OA (n=345)	TPE: STepped Exercise Program for patients with Knee OsteoArthritis (STEP-KOA)	Arthritis education	TPE group: greater disability reduction at 9 months: −6.8 points (95% CI −10.5, −3.2)
Bezalel [61]	2010	Knee OA (n=50)	TPE: Group education + self-management	Brief course in short-wave diathermy treatment	TPE group: greater disability reduction at 12 weeks (−9.0; 95%CI: −14.5, −3.4); no significant difference at 4 weeks
Ettinger [76]	1997	Knee OA (n=439)	TPE: Health education	C1: Aerobic exercise<br><br>C2: Resistance exercise	Limited improvement in disability in C1 and C2 groups compared to TPE group with an average improvement difference of 10% and 8% respectively at 18 months
Maurer [78]	1999	Knee OA (n=113)	TPE: Education + informative material	Exercise	No significant difference at 8 and 12 weeks
Nagasawa [78]	2022	Knee OA (n=30)	TPE: Acceptance and commitment therapy + exercise	Exercise	No significant difference at post-treatment and at 4 weeks
Taglietti [37]	2018	Knee OA (n=60)	TPE: Education group	Aquatic exercise	Significant difference in favor of aquatic exercise group at 8 weeks (MD −14.2; CI 95% −18, −10.5), with an effect reduction at 3 months (MD −12.3; CI 95% −24.6, −6.1)

FIRST AUTHOR	YEAR	POPULATION (OSTEOARTHRITIS)	INTERVENTION	COMPARISON	RESULTS (DISABILITY)

FIRST AUTHOR	YEAR	POPULATION (KNEE PAIN)	INTERVENTION	COMPARISON	RESULTS (DISABILITY)
James [77]	2021	Patello-femoral pain (n=24)	TPE: Education + exercise	Exercise	Both groups improved at post-treatment, with no significant difference

Legend of acronyms. OA (Osteoarthritis); STEP-KOA (STepped Exercise Program for patients with Knee OsteoArthritis); TPE (Therapeutic Patient Education)

Table S9. Results of included studies on quality of life.

FIRST AUTHOR	YEAR	POPULATION (OSTEOARTHRITIS)	INTERVENTION	COMPARISON	RESULTS (QUALITY OF LIFE - QOL)
Ackerman [81]	2012	Hip OA, Knee OA (n=126)	TPE: Arthritis self-management program	Arthritis self-help book	No between-group difference at 12 months (AQOL score for control group at 12 months=0.61 (95% CI 0.55 to 0.67), compared to 0.59 (95% CI 0.51 to 0.68) for TPE group
Hopman-Rock [70]	2000	Hip OA, Knee OA (n=105)	TPE: Self-management program	Waitlist	No significant difference at 6 months follow-up
Saffari [82]	2018	Hip OA, Knee OA (n=120)	TPE: Educational cognitive behavioral therapy program	Usual care	Greater improvement in all SF-12 subscales for the TPE group at 3 months ($p < 0.01$) except the vitality subscale, compared to usual care. The EQ-5D-3L and EQ-VAS improved significantly within both groups, but more in the TPE group at 3 months ($p < 0.01$)
Brosseau [80]	2012	Knee OA (n=222)	TPE: Cognitive behavioral therapy program + walking	C1: Walking<br>C2: Self-directed walking	No significant differences in SF-36 among the 3 groups with similar improvement at 12 and 18 months
Coleman [47]	2012	Knee OA (n=147)	TPE: Cognitive behavioral therapy	Usual care	Minimal differences in SF-36 subscales favoring the TPE group at 8 weeks; only physical function and physical body pain maintained significant results at 6 months (5.67; 95%CI 0.4, 10.93; and 6.06; 95%CI 0.04, 12.07 respectively)
Khachian [71]	2020	Knee OA (n=80)	TPE: Self-management + exercise + standard medical care	Standard medical care	TPE group: greater improvement in the self-management post-treatment at KOOS-QOL (+23.12, p<0,001) compared to control group

FIRST AUTHOR	YEAR	POPULATION (OSTEOARTHRITIS)	INTERVENTION	COMPARISON	RESULTS (QUALITY OF LIFE - QOL)
Marconcin [66]	2018	Knee OA (n=80)	TPE: Group self-management + exercise	Educational group	No significant difference in KOOS-QOL at post-treatment: SMEG - 13.7 (19.5); EC -8.2 (18.0)
Nunez [83]	2006	Knee OA (n=100)	TPE: Education and functional readaptation	Physician visit	No significant difference in SF-36 subscales at 9 months
Rezende [39]	2016	Knee OA (n=228)	TPE: Multiprofessional education program	Educational material delivery	No significant difference between group at SF-36 scale at 4 and 12 months
Rezende [38]	2017	Knee OA (n=228)	TPE: Multiprofessional education program	Educational material delivery	No significant difference between group at SF-36 scale at 1 and 2 years
Skou [59]	2015	Knee OA (n=100)	TPE: MEDIC treatment (education, exercise, insoles, weight loss and/or pain medication)	Usual care	Greater reduction in KOOS QOL subscale in the TPE group compared to the control group of -10.9 (CI95% -16.8, -5.0) at 1 year
Victor [49]	2005	Knee OA (n=193)	TPE: Educational program	Waitlist	No differences in SF-36 except for the vitality subscale at 1 year (mean difference -5.5, CI 95% -10, -0,9)

FIRST AUTHOR	YEAR	POPULATION (KNEE PAIN)	INTERVENTION	COMPARISON	RESULTS (QUALITY OF LIFE - QOL)
Jinnouchi [46]	2023	Chronic Knee Pain (n=46)	TPE: Brief education for self-management	Written educational material	No differences (EQ-5D) at 4 and 12 weeks

FIRST AUTHOR	YEAR	POPULATION (TENDINOPATHY)	INTERVENTION	COMPARISON	RESULTS (QUALITY OF LIFE - QOL)
Wilson [63]	2023	Gluteal tendinopathy (n=204)	TPE: Education + exercise	C1: Corticosteroid injection<br>C2: Wait and see	Quality of life: Mean QALYs over 1- year follow-up was greatest in the ED+EX with an Estimated QALY gains relative to CSI of 0.052 (CI95% 0.019, 0.085) and to WS of 0.057 (CI95% 0.025, 0.090)

Legend of acronyms. EQ-5D (EuroQol Five-dimensional); EQ-VAS (EuroQol Visual Analogue Scale); KOOS-QOL (Knee Injury and Osteoarthritis Outcome Score-Quality Of Life); QOL (Quality Of Life); OA (Osteoarthritis); QALY(Quality-Adjusted Life Year); SF-36 (Short Form 36 health survey questionnaire); TPE (Therapeutic Patient Education)
